# Optimization of concurrent production of xylanolytic and pectinolytic enzymes by *Bacillus safensis* M35 and *Bacillus altitudinis* J208 using agro-industrial biomass through Response Surface Methodology

**DOI:** 10.1038/s41598-020-60760-6

**Published:** 2020-03-02

**Authors:** Vihang S. Thite, Anuradha S. Nerurkar, Nandita N. Baxi

**Affiliations:** 1 Department of Microbiology and Biotechnology Centre, Faculty of Science, The Maharaja Sayajirao University of Baroda, Vadodara, Gujarat 390002 India; 2 Department of Biological Sciences and Biotechnology, Institute of Advanced Research, Koba institutional area, Gandhinagar, Gujarat 382426 India

**Keywords:** Applied microbiology, Industrial microbiology

## Abstract

Application of crude xylanolytic and pectinolytic enzymes in diverse industrial processes make these enzymes commercially valuable and demand their production process to be cost-effective. Out of four different agrowaste biomass, wheat bran (WB) and citrus peel (CP), when amended as fermentation substrates, respectively induced the highest xylanolytic enzymes and pectinolytic enzymes from both, *B. safensis* M35 and *B. altitudinis* J208. Further, the simultaneous amendment of WB and CP yielded concurrent production of these cellulase free xylanolytic and pectinolytic enzymes. Hence, the quadratic model was developed using the Central Composite Design of Response Surface Method (CCD-RSM). The model gave the concentration values for WB and CP substrates to be amended in one single production medium for obtaining two optimized predicted response values of xylanase activity and pectinase activity units, which were further practically validated for the xylanase and pectinase production responses from the optimized production medium (OPM). These practically obtained response values from OPM were found to be in accordance with a range of 95% predicted intervals (PI) values. These observations verified the validity of the predicted quadratic model from RSM and suggested that both xylanase and pectinase enzymes can be induced concurrently from both of the bacterial strains.

## Introduction

Xylanases and pectinases are the commercially important industrial groups of enzymes, members of which exhibit diverse xylanolytic and pectinolytic enzymatic activities. These groups of enzymes harbor a huge commercial potential as their biotechnological applications span broad spectra in diverse industries such as biofuels, pulp-paper, food, animal feed, textile, fiber, etc. Out of these, biofuel industries demand these xylanolytic and pectinolytic enzymes play their accessory role to the core cellulase enzymes for improving plant biomass saccharification. Whereas, animal feed industries require combination of cellulase, xylanase and or pectinase for improving the nutrition quality of grain and feed. On the other hand, pulp and paper as well as textile industries need the cellulase free xylanase and pectinase enzymes for their respective applications, i.e., to prepare hemicellulose free cellulose papers, to remove pectin (rhamnogalacturonan) coating from cotton and denim fibers, Food and beverage industries also need xylanase and pectinase enzymes to clarify pectin polymeric fraction from fruit juices and to improve the tea flavor^1,2^. These applications append the worth to the organisms which can produce the xylanase and pectinase enzymes and the cellulase free nature of such enzymes is an add-on benefit to this.

Three *Bacillus* strains M35, R31, and J208 have been isolated from camel, bull and buffalo dung samples and have been identified as *B. safensis* M35, *B. altitudinis* R31 and *B. altitudinis* J208 during our earlier work^3^. These strains have been shown to produce an endo-xylanase, and three types of endo-pectinases i.e., polygalacturonate hydrolase (PGase), pectate lyase (PL) and pectin lyase (PL). Studies on the physico-chemical properties of these enzymes during our earlier work revealed diverse characteristics along with their cellulase free feature imparting industrial importance to these enzymes^3,4^. The accessory potential of these cellulase free xylanase and pectinase enzymes to the commercial cellulase has been proven through the enzyme cocktail mediated saccharification of raw and pretreated agrowaste biomass^5,6^. As many industrial applications require both xylanase and pectinase enzymes at the same time, it would be economical and convenient to obtain both the enzymes from a single isolate in the single optimized production medium (OPM). The use of pure polysaccharide substrates as enzyme inducers in such OMP requires a preliminary purification step for these polysaccharide substrates. The current methods employed for obtaining pure xylan and pectin substrates consume time, resources and capital investments. Moreover, such purified substrates can induce only one enzyme at a time. On the other hand, agrowaste biomass, which is cheap and easily available in bulk as well as contains cellulose, hemicellulose (xylan) and rhamnogalacturonan (pectin) as its cell wall constituents, can induce more than one enzyme at a time and can become a good alternative in the production media leading to cost-effective process. In literature also, such crude agrowaste material has been used for the cost-effective production of individual enzymes. Therefore, the replacement of these pure polysaccharide substrates in the conventional medium with their inexpensive counterparts would make the enzyme production process cost-effective^7^.

There are several reports available on optimization of media components as well as physical parameters of growth conditions for the production of either xylanase or pectinase enzymes individually. Increased production of xylanase has been optimized from *B. pumilus* 3GAH and *B. mojavensis* UEB-FK in two different studies^8,9^. Optimized pectinase production from *B. licheniformis* has been reported from orange peel^10^. The reports on the optimized production of both, xylanase and pectinase enzymes, together in one single medium are very scanty for some of the *Bacillus* species in the literature. *B. pumilus* AJK was reported for concurrent production of cellulase free xylanase and pectinase enzymes on fermentation medium containing 2% of each, wheat bran and mosambi peel^11,12^. While *B. firmus* SDB9 was reported for concomitant production of xylanase, pectinase and cellulase enzymes from submerged fermentation of pectin salt medium^13^. The literature lacks the mention of *B. safensis* as well as *B. altitudinis* regarding the optimization of inducer substrate concentrations for concurrent production of cellulase free xylanolytic and pectinolytic enzymes.

Hence, the work was taken up to screen diverse crude industrial agrowaste biomass substrates for their abilities to induce xylanolytic and pectinolytic enzymes simultaneously from two different *Bacillus* isolates, i.e., *B. safensis* M35 and *B. altitudinis* R31, which is a novelty of the studies and not reported so far. For this purpose, diverse crude agrowaste biomass were used as submerged fermentation substrates and for the first time, screening and evaluation of their xylanase and/or pectinase inducing potential was based on the growth kinetic as well as enzyme production kinetic parameters for both the isolates in the present studies. The concentration of selected inducer substrate in the production medium was further optimized through central composite design (CCD) of response surface methodology (RSM) in design expert software for concurrent production of cellulase free xylanase-pectinase enzymes from M35 and J208 strains and theoretically predicted values were practically validated.

## Material and Methods

### Crude and pure polysaccharide substrates and other chemicals

Citrus peels (CP) were purchased from the local market, freeze-dried, and finely ground in a grinder. Wheat bran (WB), cotton seed cake (CSC) and rice bran (RB) were purchased from local industries (near Vadodara, Gujarat, India) and stored in an airtight container at room temperature. Molasses (M) was collected from the local market and stored at −20 °C to avoid microbial growth. These five agro-industrial residues were used as a source of crude complex polysaccharide substrates and explored for enzyme production. Whereas, Birchwood xylan (Xn, Sigma-Aldrich, Missouri, USA) and Citrus pectin (Pn, HiMedia, Mumbai, India) were used as commercially extracted pure complex polysaccharide substrates for enzyme production. Henceforth, these agro-industrial residues and commercially extracted polysaccharides will be referred to as crude and pure substrates respectively. Glucose, a pure carbon source, was used as control substrate for growth and enzyme production. All other chemicals used for media preparations and enzyme quantification assays were of analytical grade and purchased from HiMedia (Mumbai, India) or Sigma-Aldrich (Missouri, USA).

### Bacterial strains and media used for enzyme production

*Bacillus* strains *B. safensis* M35 (MCC3305) and *B. altitudinis* J208 (MCC3307) were studied for concurrent production of xylanase and pectinase enzymes. Pure cultures were maintained by inoculation and incubation on nutrient agar (NA) plates at 37 °C for 24 h followed by storage at 4–6 °C. In 250 ml Erlenmeyer flask, 100 ml of basal production medium BHM-YEP comprising (g/1000 ml) Bushnell Haas Medium (BHM) 3.27, Yeast extract (YE) 0.25 and Peptone (P) 0.75 was prepared, amended with pure or crude polysaccharide substrates (as separately mentioned in upcoming sections for individual experiments) and sterilized by autoclaving at 10 lbs for 20 min. This sterilized medium was inoculated with freshly grown 0.2 OD_600 nm_ set culture in nutrient broth (NB) and incubated at 37 °C at 160 rpm till up to 96 h and studied for the following parameters.

### Parameters analyzed

After the samples were collected, part of it was used for microbial growth measurement. The remaining sample was centrifuged at 10,000 rpm for 10 min to obtain the cell-free supernatant (CFS) and subjected to quantify secretory protein as well as xylanase and pectinase activities through colorimetric estimations on Tecan Infinite M200 Pro Nanoquant multimode reader. The obtained crude xylanase and pectinase enzymes were further referred to as M35 xylanase and J208 xylanase as well as M35 pectinase and J208 pectinase throughout the studies.

#### Growth measurement

Growth in terms of OD_600 nm_ was estimated in aliquots withdrawn from the flasks in a 24 well microtiter plate on Tecan Infinite M200 Pro using i-Control software based on normalization with uninoculated media control.

#### Protein estimation

The amount of secretory protein in CFS was estimated using Bradford method^14^. Samples were diluted properly using DW to 1.0 ml volume. 1.0 ml of Bradford reagent was added and the reaction mixture was incubated in dark for 10 min and followed by measurement of A_595 nm_. Using bovine serum albumin (BSA) as a standard the amount of protein equivalent to BSA was calculated.

#### Xylanase (xylan hydrolase) activity

Fifty microliters of appropriately diluted crude CFS from the individual isolate was added to 250 µl of 50 mM Tris-Cl pH 8.0 buffered 0.5%w/v xylan and incubated in water-bath at 40 °C for 10 min. The reaction was terminated by the addition of 300 µl dinitro salicylic acid (DNS) reagent followed by boiling in water-bath for 10 min. Once the system was cooled down to room temperature, DW was added to make volume up to 1.5 ml and A_540 nm_ was measured. One unit of xylanase activity was defined as the amount of xylanase required to release an end product equivalent to one µmol of D-xylose in reaction mixture per unit time in optimum incubation conditions^3,15^.

#### Pectinase (pectin lyase) activity

Fifty microliters of appropriately diluted crude CFS from the individual isolate was added to 250 µl of 50 mM Tris-Cl pH 8.0 buffered 0.5%w/v pectin and incubated in water-bath at 50 °C for 30 min. The reaction was terminated by adding 500 µl 1 N NaOH followed by incubation at 76 °C for 10 min. To this 600 µl of 1 N HCl followed by 500 µl of 0.04 M 2-thiobarbituric acid were added and incubated at 76 °C for 10 min. Developed pink coloration was estimated as A_550 nm_. One unit of pectin lyase activity was defined as the amount of enzyme required to increase the A_550 nm_ of the reaction mixture by 0.01 value per unit time in optimum incubation conditions^4,16^.

#### Kinetic characteristics for enzyme production

Kinetic characteristics for the enzyme production process were estimated in terms of the volumetric rate of enzyme production as well as the specific rate of enzyme production (or economic coefficient). These parameters were compared to find the best substrate for efficient enzyme production^17,18^.

- **Volumetric rate of the enzyme production process (Q**_**p**_**):** It is defined as total units of enzyme produced in one hour when the organism was grown in 1 L medium (IUlit^−1^h^−1^).
- **Specific rate of the enzyme production** or **Economic coefficient (Y**_**p/s**_**):** It is defined as total units of enzyme produced per gram of introduced substrate (IU/g^−1^).

### Effect of polymeric substrates on growth and enzyme production

Zero point five percent (v/v) inoculum of freshly grown 0.2 OD_600_ set culture was transferred to 250 ml Erlenmeyer flask containing100 ml sterilized BHM-YEP medium amended individually with 0.5% (v/v or w/v) of pure or crude fermentation substrates i.e., citrus peel (CP), cotton seed cake (CSC), molasses (M), pectin (Pn), rice bran (RB), wheat bran (WB) and xylan (Xn). Glucose (G) was used as control. This inoculated medium was incubated at 37 °C for 72 h at 160 rpm. Samples were collected after every 12 h and quantified for microbial growth, secretory protein, xylanase, and pectinase activities. Parameters of enzyme production kinetics on these substrates were evaluated.

#### Screening for enzyme induction

To discriminate between the enzyme-inducing and growth-enhancing nature of various complex substrates, a comparison for normalized growth ratio as well as normalized activity ratio for xylanase and pectinase enzymes were carried out for each isolate. Normalization was done using the values of growth, xylanase and pectinase activities obtained on individual pure or crude complex fermentation substrate (CP, CSC, M, Pn, RB, WB, and Xn) to the simple fermentation substrate Glucose (G) as mentioned below.

1 $$Xylanase\,activity\,ratio=\frac{Xylanase\,units\,obtained\,on\,polysaccharide}{Xylanase\,units\,obtained\,on\,glucose}$$

2 $$Pectinase\,activity\,ratio=\frac{Pectinase\,units\,obtained\,on\,polysaccharide}{Pectinase\,units\,obtained\,on\,glucose}$$

3 $$Growth(O{D}_{600})\,ratio=\frac{Growth\,(O{D}_{600})\,obtained\,on\,substrate}{Growth\,(O{D}_{600})\,obtained\,on\,glucose}$$

### Cumulative effect of inducer substrates on enzyme production

To study the cumulative effect of selected inducer substrates on *B. Safensis* M35 and *B. altitudinis* J208 for concurrent production of xylanases and pectinases, 0.5%w/v of each, CP, and WB were amended simultaneously in BHM-YEP media and all the parameters were estimated as mentioned in Section 2.3.

### Optimization of inducer substrate concentration on enzyme production

In order to determine the optimum concentration of inducer substrate(s) in the production medium for concurrent production of xylanolytic and pectinolytic enzymes from *Bacillus* isolates, the relationship between the experimental independent variable factors i.e., concentration of inducer substrates WB (F_1_) and CP (F_2_) to obtain the maximum response of xylanase (R_1_) and pectinase (R_2_) from each of M35 and J208 was analyzed through central composite design (CCD) using response surface methodology (RSM) which follows the second-order polynomial equation and allows to develop up to a cubic model^19^. In the present study, Design Expert Software (Version 7.1.6, State-Ease, Minneapolis, USA) was used to design and analyze the experiments containing a set of 11 runs. BHM-YEP medium (100 ml) was prepared, supplemented with different WB and CP concentrations (F_1_ and F_2_ as designed by software) in 250 ml Erlenmeyer flask and autoclaved. After transferring 0.5% v/v inoculum from freshly grown 0.2 OD_600_ set culture, media was incubated at 37 °C up to 72 h at 160 rpm. Samples were collected and subjected to estimations of xylanase and pectinase production responses as mentioned earlier in this section.

### Validation of optimized substrate concentrations for concurrent production of xylanase-pectinase enzymes

One hundred milliliters of optimized production medium (OPM) was prepared using basal production medium BHM-YEP amended with optimized concentrations of WB and CP in 250 ml Erlenmeyer flask and autoclaved. After transfer of 0.5% (v/v) inoculum from freshly grown 0.2 OD_600_ set culture, media was incubated at 37 °C up to 72 h at 160 rpm. After every 12 h, samples were collected and subjected to estimations as mentioned in section 2.3.

### Data analysis

All quantitative estimation experiments were performed individually in triplicates (n = 3) and result data along with error values are represented as Mean ± Standard Error of Mean (SEM) for each experiment either in GraphPad Prism 6.0 or Origin 2017 software. Statistical analysis was carried out using the Two-way ANOVA method in GraphPad Prism 6.0. Design-Expert Software version 7.0 was used to perform response surface studies using Central Composite Design for enzyme inducer optimization and to produce the contour graphs.

## Results and Discussion

### Effect of polymeric substrates on growth and enzyme production

**Figure 1:**
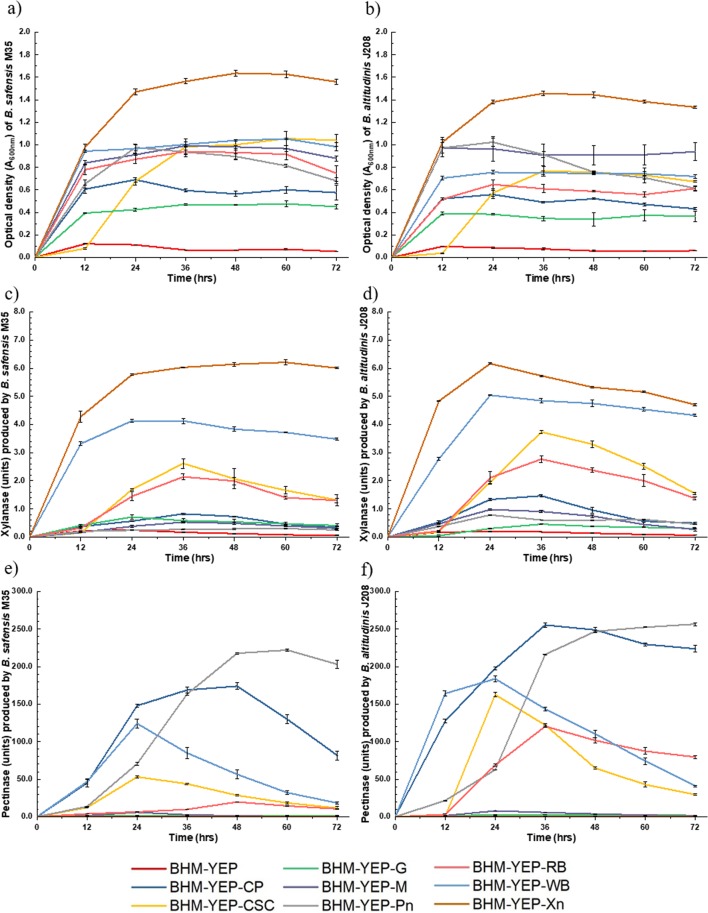
Growth of *Bacillus* strains and production of xylanolytic and pectinolytic enzymes on BHM-YEP medium with and without supplementation of individual substrates. Growth of (**a**) *B. safensis* M35 and (**b**) *B. altitudinis* J208; Xylanase activity from CFS of (**c**) *B. safensis* M35 and (**d**) *B. altitudinis* J208; Pectinase activity from CFS of (**e**) *B. safensis* M35 and (**f**) *B. altitudinis* J208 - observed up to 72 h on BHM-YEP media supplemented individually with citrus peel (CP), cotton seed cake (CSC), glucose (G), molasses (M), pectin (Pn), rice bran (RB), wheat bran (WB) and xylan (Xn). Values plotted are Mean ± Standard Error of Mean (SEM) for n = 3.

Growth of *B. safensis* M35 and *B. altitudinis* J208 in terms of OD_600 nm_ is depicted in Fig. 1a,b respectively. In comparison with basal BHM-YEP medium alone or basal BHM-YEP amended with glucose (G), supplementation of each polysaccharide substrate to basal BHM-YEP individually resulted in increased growth for both the isolates. Among all supplemented substrates the least growth was observed on glucose (G) supplementation throughout the incubation period. The growth further increased in this order, citrus peel (CP), rice bran (RB), cotton seed cake (CSC), molasses (M), wheat bran (WB), pectin (Pn) and resulted in highest on supplementation of xylan (Xn). Only CSC supplementation was earmarked for the late stationary phase at ~36 h with extended lag and log phase.

Figure 1c,d represent xylanase activity units and Fig. 1e,f represent pectinase activity units exhibited by *Bacillus* strains M35 and J208 respectively on BHM-YEP medium with and without carbohydrate supplementations. Commercial beechwood xylan (Xn) and crude substrate WB, when individually amended in media, resulted in 6–8 units and 3.5–5 units of xylanase activities respectively within 12 h of incubation. Pectinase activities were detected within 12 h of incubation from WB and CP supplemented media, and later, after 48 h of incubation, commercial citrus pectin (Pn) supplemented media exhibited the highest pectinase activities in the range of 220–280 units. Supplementation of CSC and RB resulted in moderate activities of xylanase after 36 h and pectinase after 24 h of incubation. In literature also *Streptomyces* sp. QG-11-3 has been reported to produce xylanase and pectinase enzymes after supplementing Horikoshi medium supplemented with diverse crude agrowaste i.e., wheat bran, rice bran, cottonseed cake^20^.

Thus, it can be stated that all these crude and commercial polysaccharide substrates supported the luxuriant growth of bacterial isolates during the submerged fermentation process, but only some of them could support the xylanase and/or pectinase production. Molasses (M) and glucose (G) yielded negligible xylanase and pectinase enzymes. WB, CSC, and RB could concurrently produce both the xylanases and pectinases. In literature, several spp. of bacteria and fungi such as *Aureobasidium pullulans Y-2311-1, Bacillus* sp. DT7, *Aspergillus niger* LB-02-SF, *Streptomyces* sp. P12–137, have been used for the separate production of xylanase and pectinase enzymes from such crude polysaccharide substrates^21–24^. Thus, it was necessary to screen these substrates for efficient concurrent induction of xylanase and pectinase enzymes.

### Screening for better enzyme inducer substrate(s)

During the balanced growth, an increase in the biomass is accompanied by a comparable increase of all other measurable properties of the population, e.g., protein, RNA, DNA, and intracellular water, etc. During the balanced growth, components such as DNA, RNA, and proteins, which are being synthesized by the cells, increase at the same rate as cell number. Based on this phenomenon, it becomes easy to measure any of these components and determine the growth rate, either of which is dependent on the initial population used for inoculation^25,26^. Thus, in the normal condition of balanced growth, a cell produces an enzyme at a constant rate which is directly proportional to the cell number and growth rate. But the incorporation of the inducer compounds in the media results in the induced production of the enzymes by each of the bacteria. Hence, the amount of the enzyme produced per bacterium rises and subsequently levels off a value determined by the growth rate^27^.

**Figure 2:**
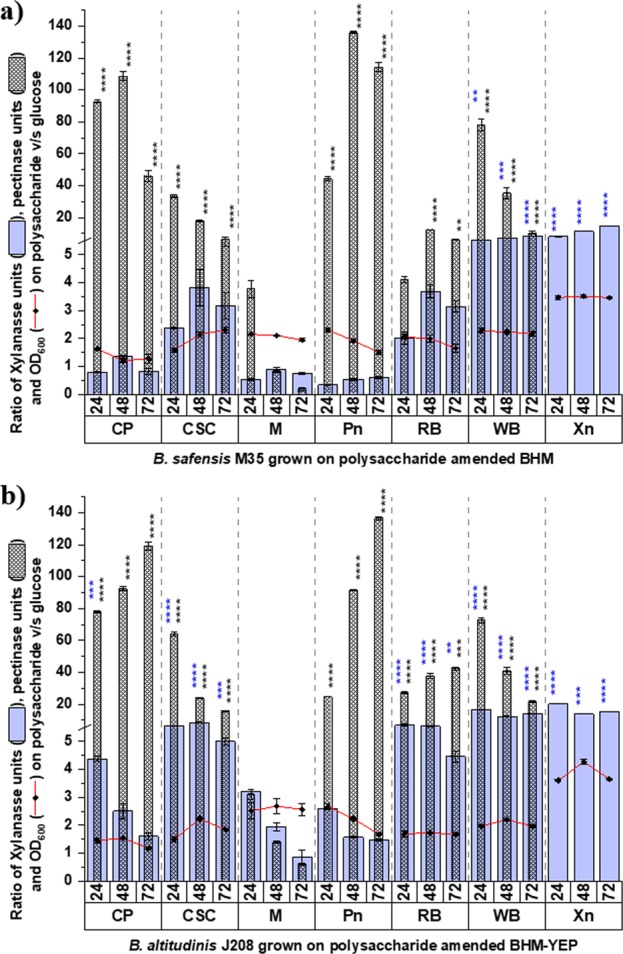
Screening of substrates for induction of xylanases and pectinases individually. Comparison of normalized growth ratio with normalized activity ratio of xylanase (blue column) as well as pectinase (dotted column) for (**a**) *B. safensis* M35 and (**b**) *B. altitudinis* J208; Values plotted are Mean ± Standard Error of Mean (SEM) for n = 3; Asterisk marks depicts significant difference between xylanase (blue coloured *) as well as pectinase (black coloured *) activity ratio from growth ratio (* = *p* < 0.05, ** = *p* < 0.01, *** = *p* < 0.001 and **** = *p* < 0.0001).

As the inoculum was constant for each culture, the normalized ratio of microbial growth, xylanase, and pectinase activities on complex substrate v/s simple substrate were compared to screen a better enzyme inducer substrate(s). Figure 2a,b represent such comparison for *B. safensis* M35 and *B. altitudinis* J208 respectively. Despite higher growth ratio on Xn and Pn supplemented media, significantly higher xylanase and pectinase activity ratios (*p* < 0.0001) from the respective media suggested that both of these commercial polysaccharide substrates, i.e. Xn and Pn, are good growth enhancers as well as xylanase and pectinase inducers respectively. CP exhibited significant difference for pectinase ratio making it a good pectinase inducer while WB exhibited a significant difference for both xylanase and pectinase suggesting it as a good inducer for both the enzymes. CSC and RB exhibited pectinase and xylanase ratios significantly higher than growth ratios suggesting that these substrates served as moderate to good xylano-pectinolytic enzyme inducers. In literature, *B. subtilis* and *B. megaterium* have been reported to exhibit xylanase production from the molasses amended media^28^. In present studies, molasses served as growth substate as it enhanced the growth of strains *B. safensis* M35 and *B. altitudinis* J208, but it could not induce the xylanase and pectinase production from the isolates.

### Enzyme production kinetics on individual plant polysaccharide substrates

Comparison of volumetric and specific rate of xylanase and pectinase production for diverse media supplementation to select the better inducer substrate(s) out of the four crude substrates (CP, CSC, RB, and WB) along with two commercial substrates (Pn and Xn) for concurrent production of xylanase and pectinase by *Bacillus* strains M35 and J208 has been shown in Table 1. The comparison suggested that commercially purified xylan and pectin are the best inducers for xylanase and pectinase respectively. Crude WB and CP induced the highest xylanase and pectinase enzymes respectively among all crude substrates. Moreover, WB also induced pectinase higher than RB and CSC. The richness of pectin in composition makes CP the best pectinase inducer. Whereas, the richness of arabinoxylan in the composition attributes in making WB as the best inducer substrate for xylanase production. Moreover, the presence of glucan-galactan also contributes to pectinase induction by WB which was higher than RB and CSC^29–32^. Although CSC and RB supported production of xylanolytic and pectinolytic enzymes, their production rates were comparatively lower than WB and CP amongst crude polysaccharides. Moreover, the cost for commercial preparation of xylan and pectin becomes a major limiting factor and hence, CP and WB were the two crude polysaccharide substrates selected for further optimization studies for concurrent production of xylano-pectinolytic enzymes.

**Table 1:** Volumetric rate and Specific rate of enzyme production on different inducer substrates.

BHM-YEP media		Volumetric rates (IU*L^−1^h^−1^)				Specific rates (IU/g substrate)			
		For xylanase production		For pectinase production		For xylanase production		For pectinase production	
		*B. safensis* M35	*B. altitudinis* J208	*B. safensis* M35	*B. altitudinis* J208	*B. safensis* M35	*B. altitudinis* J208	*B. safensis* M35	*B. altitudinis* J208
Supplemented with	CP	4.7 ± 0.9	6.9 ± 0.8	22718.0 ± 2376.3	62182.8 ± 1726.5	68.0 ± 13.4	99.2 ± 11.7	327139.7± 34219.0	895432.2 ± 24861.0
	CSC	18.3 ± 3.8	21.5 ± 0.8	3256.8 ± 888.4	8195.1 ± 393.7	263.5 ± 54.9	309.3 ± 11.5	46897.8 ± 12792.3	118008.9 ± 5669.6
	Pn	3.5 ± 0.2	6.4 ± 0.2	56437.0 ± 1955.6	71321.3 ± 782.0	50.8 ± 2.9	91.8 ± 2.9	812693.3 ± 28160.1	1027026.7 ± 11260.6
	RB	18.1 ± 1.7	19.1 ± 1.1	2948.8 ± 269.3	4117.3 ± 712.8	260.5 ± 23.8	275.4 ± 16.3	42462.3 ± 3878.1	98488.9 ± 10264.6
	WB	48.3 ± 0.9	60.1 ± 0.9	5091.1 ± 693.3	11343.8 ± 391.3	695.8 ± 13.5	865.0 ± 12.4	73312.3 ± 9984.1	183351.1 ± 5634.3
	Xn	83.4 ± 0.6	65.3 ± 0.9	0.0 ± 0.0	0.0 ± 0.0	1201.0 ± 8.6	940.8 ± 13.0	0.0 ± 0.0	0.0 ± 0.0

Values presented are Mean ± SEM (Standard Error of Mean) for n = 3.

### Screening of effective concentration range of WB and CP for enzyme production

In a separate experiment, a broad concentration range (0.5–10.0% w/v) of WB and CP was individually supplemented to basal production medium and the effective range of concentration for WB and CP was figured out to optimize the production of xylanase as well as pectinase enzymes. A bell shape pattern was clearly visible for xylanase as well as pectinase production as an effect of different concentrations of WB (Supplementary Fig. 1a,b respectively). After reaching a maximum value at 2% WB concentration, both the activities started decreasing and this decrease was significant over 3% concentration of WB making it a limiting concentration for the concurrent production of enzymes. Similarly, a bell-shaped pattern was clearly visible for xylanase and pectinase production as an effect of different concentrations of CP (Supplementary Fig. 1c,d respectively). Unlike WB, different effects of CP were earmarked on the induction of xylanase and pectinase. Xylanase was produced at all CP concentrations (maximum activity at 5%) but not pectinase. Lower concentration (up to <2%) induced pectinase activity (maximum at 1%) but above 2% pectinase production ceased making it a limiting factor for the concurrent production of enzymes. These results pointed towards the use of narrow range (0.05–3.0%) of both substrates during media optimization for obtaining concurrent induction of xylanase and pectinase enzymes.

### Effect of simultaneous supplementation of WB and CP on concurrent xylanase and pectinase production

During the simultaneous supplementation of WB and CP in BHM-YEP, their cumulative effects on concurrent production of xylanase and pectinase are shown in Supplementary Fig. 2. This simultaneous supplementation significantly increased xylanase and pectinase enzyme production when compared with individually amended WB and CP substrates (*p* < 0.0001). Moreover, increased volumetric and specific rates of enzyme production in case of simultaneous supplementation are earmarked than individual supplementation of WB and CP from the data in Table 2. In literature, *B. pumilus* AJK has been reported for the production of xylanase and pectinase after growing on 2% WB and 2% Mosambi Peels^11^. Enhancement of pectinase activities by *Aspergillus niger* LB-02-SF has been reported after the supplementation of wheat bran to the orange residues containing media^23^.

**Table 2:** Volumetric rate and Specific rate of enzyme production on WB, CP and their combination.

BHM-YEP medium		Volumetric rates (IU*L^−1^h^−1^)				Specific rates (IU/g substrate)			
		For xylanase production		For pectinase production		For xylanase production		For pectinase production	
		*B. safensis* M35	*B. altitudinis* J208	*B. safensis* M35	*B. altitudinis* J208	*B. safensis* M35	*B. altitudinis* J208	*B. safensis* M35	*B. altitudinis* J208
Supplemented with	0.5% WB	91.0 ± 4.6	103.3 ± 2.0	48100.4 ± 1931.2	79601.0 ± 3856.4	436.9 ± 22.0	496.1 ± 9.8	230881.7 ± 9269.9	382084.6 ± 18510.9
	0.5%WB + 0.5% CP	113.2 ± 4.2	116.1 ± 2.3	109877.8 ± 5520.9	198742.6 ± 14288.5	543.5 ± 20.2	557.5 ± 11.1	527413.3 ± 26500.3	953964.4 ± 68584.9
	0.5% CP	6.6 ± 0.3	16.2 ± 1.5	63029.6 ± 3013.7	146573.9 ± 829.9	31.5 ± 1.3	77.7 ± 7.3	302542.2 ± 14466.0	703554.7 ± 3983.5

Values presented are Mean ± (Standard Error of Mean) for n = 3.

### Statistical optimization of CP and WB concentration using CCD-RMS

Effect of the experimental variables (i.e., different concentrations of both inducer substrates WB and CP) and their interactions on the responses (i.e., induction of xylanase and pectinase enzymes) was calculated by the second-order polynomial equation for response surface method (RSM) using CCD. The optimum concentration for two inducers i.e., WB and CP each was determined in basal production medium for concurrent production of xylanase and pectinase from *B. safensis* M35, *B. altitudinis* J208. Table 3 represents the CCD of 11 randomized runs with corresponding xylanase and pectinase production responses by the two isolates. As can be seen from the table, standard run orders 9 to 11 (center point type) with 1.525% WB, 1.525% CP combination, exhibited a maximum 14.51 units of xylanase, and 372.47 units of pectinase activities for *B. safensis* M35. The highest values of xylanase activities for *B. altitudinis* J208 were observed was 14.90 units from 2.568% WB and 0.482% CP concentrations in std order 2 (corner point type). Whereas, the highest values of pectinase activities for *B. altitudinis* J208 were found to be 751.02 units from 1.535% WB and 1.525% CP concentrations in std order 9–11 (center point type).

**Table 3:** CCD-RSM design to study the effects of WB and CP concentrations for concurrent production of xylanase and pectinase activities.

Standard Order	Run Order	F_1_:WB (g/100 ml)	F_2_:CP (g/100 ml)	*B. safensis* M35		*B. altitudinis* J208	
				R_1_:X	R_2_:P	R_1_:X	R_2_:P
1	6	0.482	0.482	5.43	138.47	4.97	328.09
2	8	2.568	0.482	8.37	158.06	14.90	407.44
3	11	0.482	2.568	0.64	0.00	0.53	0.00
4	5	2.568	2.568	0.90	43.57	0.90	94.89
5	9	0.050	1.525	0.86	0.00	0.76	3.98
6	4	3.000	1.525	4.77	44.07	6.51	129.06
7	7	1.525	0.050	8.46	188.78	14.53	309.19
8	2	1.525	3.000	0.72	11.94	0.58	19.35
9	10	1.525	1.525	14.43	372.47	8.92	648.66
10	1	1.525	1.525	13.96	356.58	9.30	671.51
11	3	1.525	1.525	14.51	361.04	9.68	751.02

F1:WB Factor 1- Wheat Bran; F2:CP = Factor 2 - Citrus Peel; R1:X = Response 1- Xylanase activity units; R2:P = Response 2 - Pectinase activity units.

#### Model analysis

During the analysis of response data, the suitable model was selected through a fit summary report using design expert software (DX7). For xylanase and pectinase production response from each of the two isolates, Fit summary plots, ANOVA and model diagnostic plots analyses suggested that the quadratic model fits with each of these four responses (Supplementary Data). For all cases, the quadratic model was significantly above the 99.96% confidence level with *p*-value < 0.0004. Similarly, the Lack of Fit F-value for the quadratic model was >0.11 suggesting that the Lack of Fit was not significant. Further, for the quadratic model, values of correlation coefficient R^2^ as well as adjusted R^2^ values were above 0.92 and predicted R^2^ values were above 0.77 for all four responses. The thumb rule that all R^2^ should be >0.7 and the difference between the predicted R^2^ and adjusted R^2^ should not be >0.2 was followed here indicating a good correlation between the predicted and experimental R^2^. Predicted Residual Error Sum of Squares (PRESS) is a measure of how a model fits into each design point. PRESS values for the chosen model were observed to be less when compared for other models under consideration in case of all four-response data as per the rules. Adequate precision is a measure of a signal to noise ratio and its value above 4 indicates a preference for the model. Further, ANOVA analysis for selected factors and their interactions in a quadratic model of each response were carried out and they were found to be highly significant with confidence interval >99.93 indicating the significance of the model terms. Further detailed explanations on the above-mentioned terms for each of the four responses have been provided in separate analysis data in Supplementary Data. Based on this information, the effects of inducer substrates and their interactions on responses of xylanase and pectinase enzyme production were analyzed further.

#### Response surface plot for xylanase and pectinase responses

**Figure 3:**
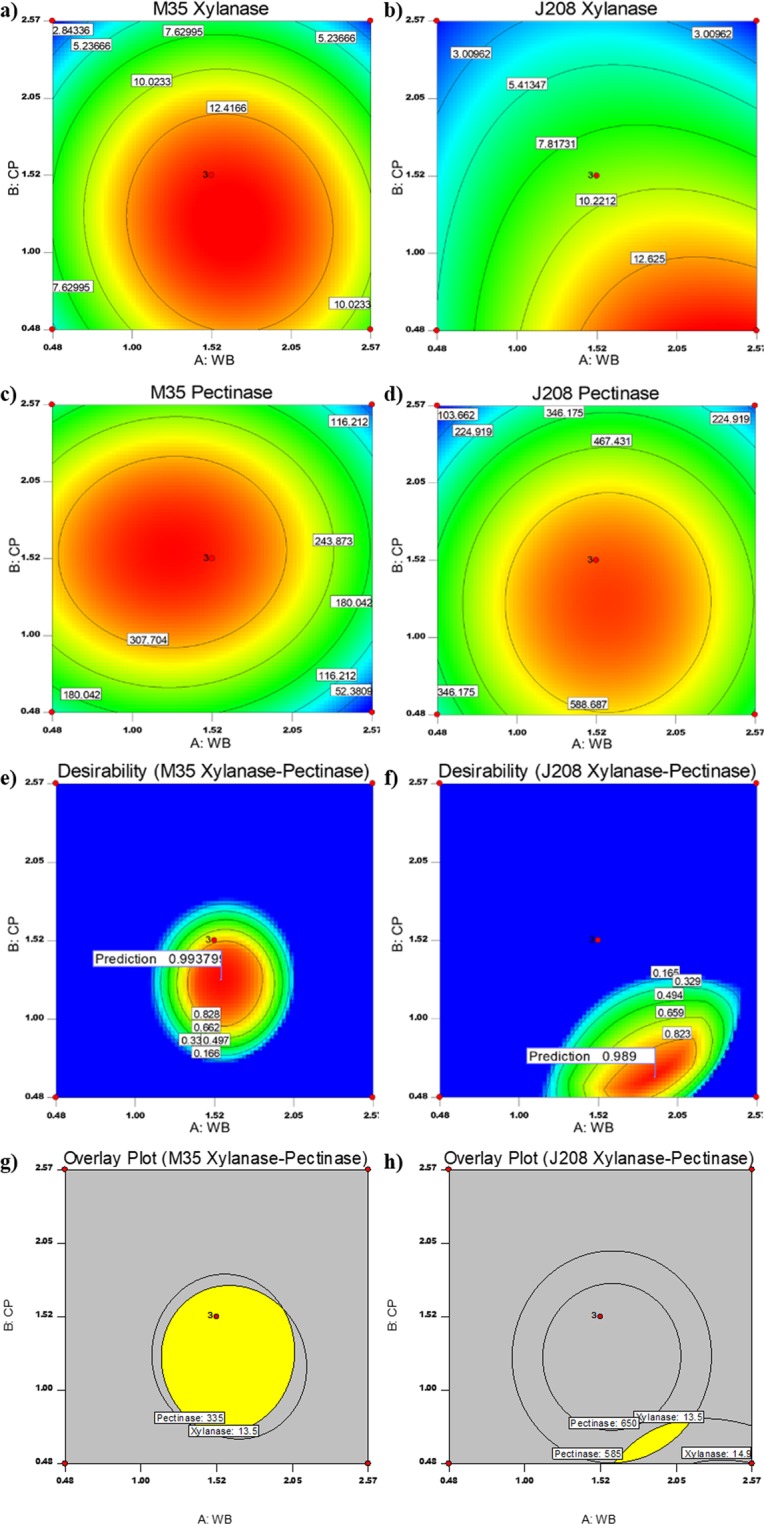
Contour plots, Desirability plots and Overlay pots for response surface prediction. (**a**–**d**) Contour plots exhibiting effects of WB and CP on xylanase production responses from (**a**) *B. safensis* M35 and (**b**) *B. altitudinis* J208; pectinase production responses from (**c**) *B. safensis* M35 and (**d**) *B. altitudinis* J208. The values represented in each box beside the contour line represent the respective enzyme activity units and are Mean for n = 3; (**e**–**h**) Desirability and Overlay plots for optimized responses of xylanase and pectinase production. X and Y-axis represents concentration (in % w/v) of WB and CP respectively; For desirability plots of (**e**) *B. safensis* M35 and (**f**) *B. altitudinis* J208, value in each box beside contour line represents the desirability value obtained through numerical optimization; For overlay plots of (**g**) *B. safensis* M35 and (**h**) *B. altitudinis* J208, value in large box represents the predicted maximum responses at mentioned variable concentrations obtained through graphical method; Yellow-coloured region in overlay plots (**g**,**h**) indicates the range of predicted highest response.

Contour plots of xylanase and pectinase production responses on WB and CP variables from two *Bacillus* strains M35 and J208 are presented in Fig. 3(a–d) respectively. The highest xylanase and pectinase response values were traced through axis adjustments for each contour plot. It was observed that moderate concentration of WB (~1.6 g) and CP (~1.2 g) yielded the highest xylanase response from *B. safensis* M35 (Fig. 3a), whereas high WB (~2.3 g) and low CP (~0.5 g) concentrations yielded the highest xylanase responses from both *B. altitudinis* J208 (Fig. 3b). But, the moderate concentration of WB (~1.5–1.6 g) and CP (~1.2–1.3 g) yielded the highest pectinase response from both isolates (Fig. 3c,d). Thus, the combination of polysaccharides containing raw agrowaste biomass such as CP and WB in combinations can induce the production of xylano-pectinolytic enzymes in one single setup which can relieve the production cost. Further, to achieve the production of both in an optimum amount, the point prediction analysis was performed.

#### Desirability plots and point prediction

Initially numerical as well as graphical optimization for xylanase and pectinase production responses were carried out in Design-Expert software through desirability plots and based on this further point prediction was performed. Numerical optimization was performed with the same range of WB and CP concentrations which was used in CCD experiments for maximized production response of xylanase and pectinase. This analysis generated desirability plots for combined production of xylanase and pectinase where desirability values approaching 1.0 were most preferable for concurrent xylanase and pectinase production (Fig. 3e,f). Analysis through axis adjustments of each contour plots suggested desirability values 0.99 and 0.98 for M35 and J208 respectively. The corresponding concentrations of WB and CP variables for X and Y axis values at the highest desirability value, on the combination, can induce the maximum xylanase and pectinase production response. The region of highest desirability values was observed in the range of variables which produced 90–100% of maximum response observed during CCD studies. And hence, 90–100% of maximum response obtained was considered as the margin of xylanase and pectinase production response for graphical optimization of media and the overlay plots for both responses were generated. As shown in Fig. 3g,h, the limits of both responses were marked as contours in graphs. Both xylanase and pectinase production responses for each isolate were overlapped. The overlapped graphs of both responses for individual isolates are shown in Fig. 3g,h and the yellow-colored region from the overlapped graphs corroborated with the region of highest prediction. From this analysis concentration values of WB and CP were predicted and validated further.

#### Validation of predicted points

**Figure 4:**
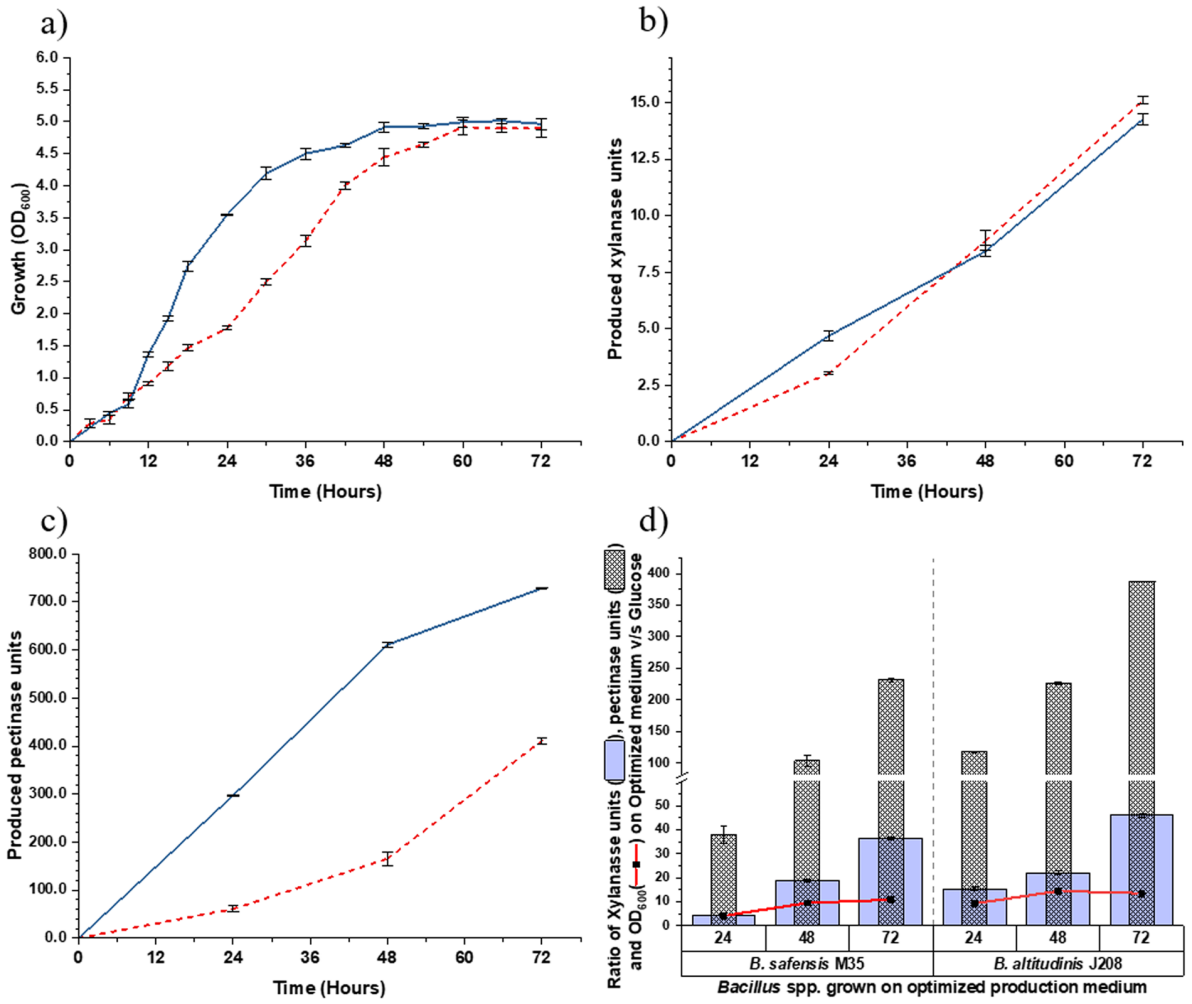
Validation of predicted concurrent enzyme production responses by selected *Bacillus* strains and their growth on optimized production medium. Observation of **(a)** Growth **(b)** Xylanase activity and **(c)** Pectinase activity. From *B. safensis* M35 (dotted line), *B. altitudinis* J208 (intact line); **(d)** Comparison of normalized growth ratio (red line) with normalized xylanase activity ratio (blue column) as well as normalized pectinase activity ratio (dotted column) for optimized production media (OPM). Values plotted are Mean ± Standard Error of Mean (SEM) for n = 3.

The predicted values of F1 and F2 for production media to be validated along with the predicted responses R1 and R2 are listed in Table 4 along with other incubation parameters. Figure 4 represents the growth as well as xylanase and pectinase activities obtained from optimized production media (OPM) along with their normalized ratios calculated as mentioned in Materials and Methods section A comparison of OD_600_ and growth ratios of OPM (Fig. 4d) with those on WB and CP containing media (Fig. 2) revealed that the OPM enhanced. the growth of bacterial isolates several folds than individual WB or CP containing medium. Further comparison of xylanase and pectinase ratios with growth ratios confirmed that though the OPM acted as a growth medium, the xylanase ratio was 3 to 4 times more than the growth ratio and the pectinase ratio was 25–30 times more than the growth ratio. The observed experimental values for xylanase and pectinase production responses during validation studies were 15.10 U (M35-xylanase), 14.27 U (J208-xylanase), 411.58 U (M35-pectinase) and 728.74 U (J208-pectinase). As shown in Table 4, all these four responses were found to be in the range of predicted interval (at 95% PI) as given by the software. These results verified the validity of the model and the existence of the optimal points indicating the adequacy of the RSM data. Co-production of xylanase (4.31 U/mL) and alkaline protease (3.66 U/mL) by *Bacillus* spp have been optimized under submerged fermentation from WB^33^.

**Table 4:** Validation of predicted points from the selected quadratic model.

Media composition and incubation conditions		Organisms	
		*B. safensis* M35	*B. altitudinis* J208
Media components (g/100 ml)	BHM	0.327	0.327
	Yeast Extract	0.025	0.025
	Peptone	0.075	0.075
**Inducer substrate (g/100 ml)**	**WB**	**1.57**	**1.90**
	**CP**	**1.26**	**0.62**
Physical parameters for incubation	Initial pH	Default (5.8)	Default (6.2)
	Temperature (°C)	37	37
	Agitation rate (rpm)	160	160
Inoculum (%v/v)		0.5	0.5
**Response 1 Xylanase**	95% PI Low	*13.38*	*12.49*
	Prediction	14.77	13.94
	95% PI High	*16.16*	*15.39*
	**Observed**	**15.01**	**14.27**
**Response 2 Pectinase**	95% PI Low	*324.44*	*376.16*
	Prediction	371.54	601.01
	95% PI High	*418.64*	*823.84*
	**Observed**	**411.58**	**728.74**

Observed response values presented as Mean, for n = 3.

## Conclusion

Among a variety of agro-industrial waste biomass, screened for their potential to induce these xylano-pectinolytic enzymes, CP induced pectinase and WB induced xylanase as well as pectinase. Therefore, the study demonstrates a novel approach where different concentrations of WB and CP were optimized in one single media based on a quadratic model developed through CCD-RSM for concurrent production of cellulase free xylanase and pectinase enzymes from strains *B. safensis* M35 and *B. altitudinis* J208 individually. In the case of, *B. safensis* M35, the productivity of the OPM (containing 1.57% WB and 1.26% CP) was found to be 15.10 U for xylanase and 411.58 U for pectinase enzymes. Whereas, in the case of *B. altitudinis* J208, the productivity of the OPM (containing 1.9% WB and 0.62% CP) was 14.27 U of xylanase and 728.74 U of pectinase enzymes. The present studies can further be extended to the optimization of other physicochemical parameters like inoculum size, C and N source, pH of media, incubation conditions like temperature, aeration speed (rpm), and incubation time and can be scaled up at bioreactor level to validate the commercial practicability of the process.

### Ethical statement

This article does not contain any studies with human participants or animals performed by any of the authors.

## Supplementary information

Supplementary Information.

## References

[1] Kumar R, Wyman CE (2009). Effect of xylanase supplementation of cellulase on digestion of corn stover solids prepared by leading pretreatment technologies. Bioresour. Technol..

[2] Rebello S (2017). Recent advancements in the production and application of microbial pectinases: an overview. Rev. Environ. Sci. Biotechnol..

[3] Thite VS, Nerurkar AS (2015). Xylanases of *Bacillus* spp. isolated from ruminant dung as potential accessory enzymes for agro-waste saccharification. Lett. Appl. Microbiol..

[4] Thite VS, Nerurkar AS (2018). Physicochemical characterization of pectinase activity from *Bacillus* spp. and their accessory role in synergism with crude xylanase and commercial cellulase in enzyme cocktail mediated saccharification of agrowaste biomass. J. Appl. Microbiol..

[5] Thite, V. S. & Nerurkar, A. S. Crude Xylanases and Pectinases from *Bacillus* spp. Along with Commercial Cellulase Formulate an Efficient Tailor-Made Cocktail for Sugarcane Bagasse Saccharification. *BioEnergy Res*. 10.1007/s12155-019-10050-5 (2019).

[6] Thite, V. S. & Nerurkar, A. S. Valorization of sugarcane bagasse by chemical pretreatment and enzyme mediated deconstruction. *Sci. Rep*. **9**, (2019). 10.1038/s41598-019-52347-7 PMC6828687 31685856

[7] Gupta A, Verma JP (2015). Sustainable bio-ethanol production from agro-residues: A review. Renew. Sustain. Energy Rev..

[8] Kallel F (2016). Statistical optimization of low-cost production of an acidic xylanase by *Bacillus mojavensis* UEB-FK: Its potential applications. Biocatal. Agric. Biotechnol..

[9] Kaur A, Singh A, Patra AK, Mahajan R (2016). Cost-effective scouring of flax fibers using cellulase-free xylano-pectinolytic synergism from a bacterial isolate. J. Clean. Prod..

[10] Bibi N, Ali S, Tabassum R (2016). Statistical Optimization of Pectinase Biosynthesis from Orange Peel by Bacillus licheniformis Using Submerged Fermentation. Waste and Biomass Valorization.

[11] Kaur A, Singh A, Dua A, Mahajan R (2017). Cost-effective and concurrent production of industrially valuable xylano-pectinolytic enzymes by a bacterial isolate Bacillus pumilus AJK. Prep. Biochem. Biotechnol..

[12] Agrawal, S., Varghese, L. M. & Mahajan, R. A novel and cost‐effective methodology for enhanced production of industrially valuable alkaline xylano-pectinolytic enzymes cocktail in short solid‐state fermentation cycle. *Biotechnol. Prog*. 10.1002/btpr.2872 (2019). 10.1002/btpr.2872 31215769

[13] Bhagat DD, Dudhagara PR, Desai PV (2016). Statistical approach for pectinase production by *Bacillus firmus* SDB9 and evaluation of pectino-xylanolytic enzymes for pretreatment of kraft pulp. J. Microbiol. Biotechnol. Food Sci..

[14] Kruger, N. J. The Bradford Method for Protein Quantitation. in *The Protein Protocols Handbook* (ed. Walker, J. M.) 15–22 (Humana Press, 2002). 10.1385/1-59259-169-8:15

[15] Ghose TK, Bisaria VS (1987). Measurement of hemicellulase activities: Part I Xylanases. Pure Appl. Chem..

[16] Nedjma M, Hoffmann N, Belarbi A (2001). Selective and sensitive detection of pectin lyase activity using a colorimetric test: application to the screening of microorganisms possessing pectin lyase activity. Anal. Biochem..

[17] Sohail, M., Siddiqi, R., Ahmad, A. & Khan, S. A. Cellulase production from Aspergillus niger MS82: effect of temperature and pH. *N. Biotechnol*. **25**, (2009). 10.1016/j.nbt.2009.02.002 19552887

[18] 2 Kinetics of microbial growth and product formation. in *Progress in* Industrial *Microbiology* (ed. Sikyta, B.) **31**, 33–61 (Science Direct, 1995).

[19] Montgomery, D. C. *Design and analysis of experiments*. (Wiley, 2009).

[20] Beg QK, Bhushan B, Kapoor M, Hoondal GS (2000). Production and characterization of thermostable xylanase and pectinase from Streptomyces sp. QG-11-3. J. Ind. Microbiol. Biotechnol..

[21] Yegin S, Buyukkileci AO, Sargin S, Goksungur Y (2017). Exploitation of Agricultural Wastes and By-Products for Production of *Aureobasidium pullulans Y-2311-1* Xylanase: Screening, Bioprocess Optimization and Scale Up. Waste and Biomass Valorization.

[22] Kashyap DR, Chandra S, Kaul A, Tewari R (2000). Production, purification and characterization of pectinase from a *Bacillus* sp. DT7. World J. Microbiol. Biotechnol..

[23] Reginatto C (2017). Pectinase production by Aspergillus niger LB-02-SF is influenced by the culture medium composition and the addition of the enzyme inducer after biomass growth. Process Biochem..

[24] L. F., P. Andrade C. C., A. Sant M. H. (2013). A Review of Xylanase Production by the Fermentation of Xylan: Classification, Characterization and Applications. Sustainable Degradation of Lignocellulosic Biomass - Techniques, Applications and Commercialization.

[25] Stanier, R. Y., Adelberg, E. A. & Ingraham, J. L. *General microbiology*. (Cambridge University Press, 1993).

[26] Stanbury, P. F., Whitaker, A. & Hall, S. J. *Principles of Fermentation Technology*. (Joe Hayton, Butterworth-Heinemann, Elsevier Publication, 2017).

[27] Novick A, Weiner M (1957). Enzyme Induction as an All-or-None Phenomenon. Proc. Natl. Acad. Sci..

[28] Irfan M, Asghar U, Nadeem M, Nelofer R, Syed Q (2016). Optimization of process parameters for xylanase production by Bacillus sp. in submerged fermentation. J. Radiat. Res. Appl. Sci..

[29] Moongngarm A, Daomukda N, Khumpika S (2012). Chemical Compositions, Phytochemicals, and Antioxidant Capacity of Rice Bran, Rice Bran Layer, and Rice Germ. APCBEE Procedia.

[30] Pütün AE, Özbay N, Koçkar ÖM, Pütün E (1997). Fixed-bed pyrolysis of cottonseed cake: Product yields and compositions. Energy Sources.

[31] Das M, Banerjee R, Bal S (2008). Evaluation of physicochemical properties of enzyme treated brown rice (Part B). LWT - Food Sci. Technol..

[32] Nandini CD, Salimath PV (2001). Carbohydrate composition of wheat, wheat bran, sorghum and bajra with good chapati/roti (Indian flat bread) making quality. Food Chem..

[33] Limkar MB, Pawar SV, Rathod VK (2019). Statistical optimization of xylanase and alkaline protease co-production by Bacillus spp using Box-Behnken Design under submerged fermentation using wheat bran as a substrate. Biocatal. Agric. Biotechnol..

