# Supplementary material for: Optimization of concurrent production of xylanolytic and pectinolytic enzymes by Bacillus safensis M35 and Bacillus altitudinis J208 using agro-industrial biomass through Response Surface Methodology
Source: Sci Rep. 2020 Mar 2;10:3824. doi: 10.1038/s41598-020-60760-6 (PMC7052231; doi:10.1038/s41598-020-60760-6)
Supplement: Supplementary file 1 — Supplementary Information. [file 41598_2020_60760_MOESM1_ESM.docx]

**Electronic Supplementary Material**

**Title:**

**Optimization of concurrent production of xylanolytic and pectinolytic enzymes by *Bacillus safensis* M35 and *Bacillus altitudinis* J208 using agro-industrial biomass through Response Surface Methodology**

**Authors:**

Vihang S. Thite***** ^a,b^, Anuradha S. Nerurkar ^a^ and Nandita N. Baxi ^a^

**Affiliation:**

^a^ Department of Microbiology and Biotechnology Centre, Faculty of Science. The Maharaja Sayajirao University of Baroda, Vadodara, Gujarat, India. 390002.

^b^ Department of Biological Sciences and Biotechnology, Institute of Advanced Research, Koba institutional area, Gandhinagar, Gujarat, India. 382426.

**E-mail and ORCID-ID:**

Vihang S. Thite*****: [thitevihang87@yahoo.com](mailto:thitevihang87@yahoo.com); ORCID-ID: 0000-0003-4197-4656

Anuradha S. Nerurkar: [anuner26@yahoo.com](mailto:anuner26@yahoo.com); ORCID-ID: 0000-0002-6674-403

Nandita N. Baxi: [nanditabaxi@yahoo.com](mailto:nanditabaxi@yahoo.com); ORCID-ID: 0000-0002-9945-0962

*** Correspondence:**

Vihang S. Thite

Department of Biological Sciences and Biotechnology, Institute of Advanced Research, Koba institutional area, Gandhinagar, Gujarat, India. 382426.

E-mail: [thitevihang87@yahoo.com](mailto:thitevihang87@yahoo.com)

Supplementary Fig 1. Screening of effective concentration range of WB and CP substrates for enzyme production (a) xylanase and (b) pectinase activities from WB supplemented BHM-YEP medium, and (c) xylanase and (d) pectinase activities from CP supplemented BHM-YEP medium from *B. safensis* M35 () and *B. altitudinis* J208 (); Data presented as Mean ± Standard Error of Mean (SEM) for n=3.

a) b) c) d)

**Supplementary Fig 2.** **Cumulative effect of CP and WB on concurrent production of xylanase-pectinase enzymes** Comparison between media amended individually with WB () or CP () and CP+WB (). Asterisk marks indicates the significant difference between xylanase produced by WB and CP+WB as well as between pectinase produced by CP and CP+WB (*= *p* < 0.05, **= *p* < 0.01, ***= *p* < 0.001, ****= *p* < 0.0001); The values represented are Mean ± Standard Error of Mean (SEM) for n=3.

Statistical analysis and model diagnostic plots for inducer substrate optimization using CCD-RSM:

**A Effect of individual factors and their interactions on xylanase production response from different isolates:**

**A.1. M35-xylanase production:**

During sequential analysis of the response surface for M35 xylanase production, it was observed that the quadratic model fits well with the data. As shown in Table A1, the quadratic model was significant above 99.99 % confidence level with p-value < 0.0001. Similarly, the Lack of Fit F-value for quadratic model was 0.2267 suggesting that Lack of Fit was not significant, relative to the pure error and there is a 22.67% chance that a “Lack of Fit F-value” this large could occur due to noise.

The quadratic model values of correlation coefficient (R^2^), adjusted R^2^ and predicted R^2^ were 0.997, 0.993 and 0.979 respectively. Values of R^2^ as well as adjusted R^2^ were > 0.7. The predicted R^2^ was in reasonable agreement with the adjusted R^2^. PRESS value of 7.00 was also suitable as it was least in comparison to other models. All these results from Table A1 indicated that the quadratic model was the most appropriate one for analysis of xylanase production data.

Therefore, analysis of variance (ANOVA) for M35 xylanase production response was performed using the quadratic model. The values of sum of squares, mean squares, F-value, and *p*-value for model, selected factors and their interactions are given in Table A2. P values < 0.05 indicates that the model terms are significant. In this case the Model F-value of 485.54 implies the model was highly significant with p-values < 0.0001 (with confidance level > 99.99%) and there is only a 0.01% chance that a "Model F-Value" this large could occur due to noise. *p*-values less than 0.05 (at confidence level of 95%) indicates the significance of the model terms.

In this case A, B, AB, A^2^, B^2^ are significant model terms. *p*-values obtained for both individual factors, viz., A (wheat bran, WB) and B (citrus peel, CP) were 0.0012 and <0.0001 respectively suggesting that CP had more significant effect on xylanase production than WB. The *p*-value of AB factor (0.0355) suggested that the interaction between WB and CP was significant for xylanase production at 95% confidence level.

The adequate precision value (39.928) of signal to noise ratio for M35 xylanase production indicated an adequate signal and the model can be used to navigate the design space. The following equation shows fitted quadratic model in terms of actual factors:

Xylanase = -7.00033 + 17.99698*WB + 11.67553*CP – 0.61709*WB*CP – 5.24935*WB^2^ – 4.43148*CP^2^.

Table A1 Fit summary plot for M35-xylanase production obtained using CCD:

| 1. Sequential Model Sum of Squares [Type I] | | | | | | |
| --- | --- | --- | --- | --- | --- | --- |
| Source | Sum of Squares | DOF | Mean Square | F-value | *p*-value, Prob>F | Prediction |
| Mean vs Total | 485.32 | 1 | 485.32 |  |  |  |
| Linear vs Mean | 76.84 | 2 | 38.42 | 1.24 | 0.3398 |  |
| 2FI vs Linear | 1.80 | 1 | 1.80 | 0.051 | 0.8274 |  |
| **Quadratic vs 2FI** | **245.13** | **2** | **122.57** | **555.55** | **< 0.0001** | **Suggested** |
| Cubic vs Quadratic | 0.89 | 2 | 0.45 | 6.37 | 0.0832 | Aliased |
| Residual | 0.21 | 3 | 0.070 |  |  |  |
| Total | 810.20 | 11 | 73.65 |  |  |  |
| 2. Lack of Fit Tests | | | | | |  |
| Source | Sum of Squares | DOF | Mean Square | F-value | *p*-value, Prob>F |  |
| Linear | 247.86 | 6 | 41.31 | 475.52 | 0.0021 |  |
| 2FI | 246.06 | 5 | 49.21 | 566.48 | 0.0018 |  |
| **Quadratic** | **0.93** | **3** | **0.31** | **3.57** | **0.2267** | **Suggested** |
| Cubic | 0.037 | 1 | 0.037 | 0.42 | 0.5832 | Aliased |
| Pure Error | 0.17 | 2 | 0.087 |  |  |  |
| 3. Model Summary Statistics | | | | | |  |
| Source | Std. Deviation | R^2^ | Adjusted R^2^ | PredictedR^2^ | PRESS |  |
| Linear | 5.57 | 0.2365 | 0.0457 | -0.1654 | 378.63 |  |
| 2FI | 5.93 | 0.2421 | -0.0828 | -0.5134 | 491.68 |  |
| **Quadratic** | **0.47** | **0.9966** | **0.9932** | **0.9785** | **7.00** | **Suggested** |
| Cubic | 0.26 | 0.9994 | 0.9978 | 0.9916 | 2.73 | Aliased |

Table A2 ANOVA for M35-xylanase production obtained using CCD:

| ANOVA for Response Surface Quadratic Model | | | | | | |
| --- | --- | --- | --- | --- | --- | --- |
| Analysis of variance table [Partial sum of squares - Type III] | | | | | |  |
| Source | Sum of Squares | DOF | Mean Square | F-value | p-value, Prob>F |  |
| Model | 323.78 | 5 | 64.76 | 293.51 | < 0.0001 | Significant |
| A-WB | 9.51 | 1 | 9.51 | 43.11 | 0.0012 |  |
| B-CP | 67.33 | 1 | 67.33 | 305.19 | < 0.0001 |  |
| AB | 1.80 | 1 | 1.80 | 8.17 | 0.0355 |  |
| A^2^ | 184.14 | 1 | 184.14 | 834.64 | < 0.0001 |  |
| B^2^ | 131.23 | 1 | 131.23 | 594.82 | < 0.0001 |  |
| Residual | 1.10 | 5 | 0.22 |  |  |  |
| Lack of Fit | 0.93 | 3 | 0.31 | 3.57 | 0.2267 | not significant |
| Pure Error | 0.17 | 2 | 0.087 |  |  |  |
| Cor Total | 324.88 | 10 |  |  |  |  |

**A.2. J208-xylanase production:**

Through the sequential analysis of the response surface for J208 xylanase production, it was observed that the quadratic model fits well with data. As shown in Table A3, the quadratic model was significant above 99.99 % confidence level with p-value 0.0001. Similarly, the Lack of Fit F-value for quadratic model was 0.3268 suggesting that Lack of Fit was not significant, relative to the pure error and there is a 32.68% chance that a “Lack of Fit F-value” this large could occur due to noise.

The quadratic model values of correlation coefficient (R^2^), adjusted R^2^ and predicted R^2^ were 0.996, 0.992 and 0.975 respectively. Values of R^2^ and adjusted R^2^ were > 0.7 and predicted R^2^ was in reasonable agreement with the adjusted R^2^. PRESS value of 7.39 was also suitable as it was least in comparison to other models. All these results from Table A3 indicated that the quadratic model was the most appropriate one for analysis of xylanase production data.

Therefore, analysis of variance (ANOVA) for J208 xylanase production response was performed using the quadratic model. The values of sum of squares, mean squares, F-value, and *p*-value for model, selected factors and their interactions are given in Table A4. *p*-values < 0.05 indicates that the model terms are significant. In this case the Model F-value of 294.84 implies the model was highly significant with p-values <0.0001 (with confidance level > 99.99%) and there is only a 0.01% chance that a "Model F-Value" this large could occur due to noise. *p*-values less than 0.05 (at confidence level of 95%) indicates the significance of the model terms.

In this case A, B, AB, A^2^, B^2^ are significant model terms. *p*-values obtained for both individual factors, viz., A (wheat bran, WB) and B (citrus peel, CP) were <0.0001 suggesting that WB and CP both had significant effect on xylanase production. The *p*-value of AB factor (0.0002) suggested that the interaction between WB and CP was significant for xylanase production at 99.9% confidence level.

The adequate precision value (40.005) of signal to noise ratio for J208 xylanase production indicated an adequate signal and the model can be used to navigate the design space. The following equation shows fitted quadratic model in terms of actual factors:

Xylanase = -0.41634 + 13.69187*WB +1.41756*CP - 2.19675*WB*CP - 2.66641*WB^2^ - 0.86562*CP^2^.

Table A3. Fit summary plot for J208-xylanase production obtained using CCD:

| 1. Sequential Model Sum of Squares [Type I] | | | | | | |
| --- | --- | --- | --- | --- | --- | --- |
| Source | Sum of Squares | DOF | Mean Square | F-value | *p*-value, Prob>F | Prediction |
| Mean vs Total | 465.93 | 1 | 465.93 |  |  |  |
| Linear vs Mean | 224.44 | 2 | 112.22 | 12.53 | 0.0034 |  |
| 2FI vs Linear | 22.84 | 1 | 22.84 | 3.28 | 0.1132 |  |
| **Quadratic vs 2FI** | **47.56** | **2** | **23.78** | **96.31** | **0.0001** | **Suggested** |
| Cubic vs Quadratic | 0.80 | 2 | 0.40 | 2.77 | 0.2084 | Aliased |
| Residual | 0.43 | 3 | 0.14 |  |  |  |
| Total | 762.00 | 11 | 69.27 |  |  |  |
| 2. Lack of Fit Tests | | | | | |  |
| Source | Sum of Squares | DOF | Mean Square | F-value | *p*-value, Prob>F |  |
| Linear | 71.35 | 6 | 11.89 | 83.08 | 0.0119 |  |
| 2FI | 48.51 | 5 | 9.70 | 67.78 | 0.0146 |  |
| **Quadratic** | **0.95** | **3** | **0.32** | **2.21** | **0.3268** | **Suggested** |
| Cubic | 0.15 | 1 | 0.15 | 1.03 | 0.4166 | Aliased |
| Pure Error | 0.29 | 2 | 0.14 |  |  |  |
| 3. Model Summary Statistics | | | | | |  |
| Source | Std. Deviation | R^2^ | Adjusted R^2^ | PredictedR^2^ | PRESS |  |
| Linear | 2.99 | 0.7580 | 0.6976 | 0.5305 | 139.01 |  |
| 2FI | 2.64 | 0.8352 | 0.7646 | 0.6336 | 108.49 |  |
| **Quadratic** | **0.50** | **0.9958** | **0.9917** | **0.9751** | **7.39** | **Suggested** |
| Cubic | 0.38 | 0.9985 | 0.9951 | 0.9659 | 10.10 | Aliased |

Table A4. ANOVA for J208-xylanase production obtained using CCD:

| ANOVA for Response Surface Quadratic Model | | | | | | |
| --- | --- | --- | --- | --- | --- | --- |
| Analysis of variance table [Partial sum of squares - Type III] | | | | | |  |
| Source | Sum of Squares | DOF | Mean Square | F-value | p-value, Prob>F | Significant |
| Model | 294.84 | 5 | 58.97 | 238.84 | < 0.0001 | significant |
| A-WB | 42.48 | 1 | 42.48 | 172.04 | < 0.0001 |  |
| B-CP | 181.96 | 1 | 181.96 | 737.00 | < 0.0001 |  |
| AB | 22.84 | 1 | 22.84 | 92.52 | 0.0002 |  |
| A^2^ | 47.51 | 1 | 47.51 | 192.43 | < 0.0001 |  |
| B^2^ | 5.01 | 1 | 5.01 | 20.28 | 0.0064 |  |
| Residual | 1.23 | 5 | 0.25 |  |  |  |
| Lack of Fit | 0.95 | 3 | 0.32 | 2.21 | 0.3268 | not significant |
| Pure Error | 0.29 | 2 | 0.14 |  |  |  |
| Cor Total | 296.07 | 10 |  |  |  |  |

**B. Effect of individual factors and their interactions on pectinase production response from different isolates:**

**B.1. M35-pectinase production:**

During sequential analysis of the response surface for M35 pectinase production, it was observed that the quadratic model fits well with data. As shown in Table B1, the quadratic model was significant above 99.99 % confidence level with *p*-value < 0.0001. Similarly, the Lack of Fit F-value for quadratic model was 0.1544 suggesting that Lack of Fit was not significant, relative to the pure error and there is a 15.44% chance that a “Lack of Fit F-value” this large could occur due to noise.

The quadratic model values of correlation coefficient (R^2^), adjusted R^2^ and predicted R^2^ were 0.998, 0.989 and 0.963 respectively. Values of R^2^ as well as adjusted R^2^ were > 0.7. The predicted R^2^ was in reasonable agreement with the adjusted R^2^. PRESS value of 8384.31 was also suitable as it was least in comparison to other models. All these results from Table B1 indicated that the quadratic model was the most appropriate one for analysis of pectinase production data.

Therefore, analysis of variance (ANOVA) for M35 pectinase production response was performed using the quadratic model. The values of sum of squares, mean squares, F-value, and *p*-value for model, selected factors and their interactions are given in Table B2. *p*-values < 0.05 indicates that the model terms are significant. In this case the Model F-value of 2.2x10^5^ implies the model was highly significant with p-values <0.0001 (with confidance level > 99.99%) and there is only a 0.1% chance that a "Model F-Value" this large could occur due to noise. *p*-values less than 0.05 (at confidence level of 95%) indicates the significance of the model terms. In this case A, B, A^2^, B^2^ are significant model terms. *p*-values obtained for both individual factors, viz., A (wheat bran, WB) and B (citrus peel, CP) were 0.0388 and 0.0001 respectively suggesting that CP had more significant effect on pectinase production than WB. The *p*-value of AB factor (0.4860) suggested that the interaction between WB and CP was not significant for pectinase production at 95% confidence level also.

The adequate precision value 31.832 of signal to noise ratio for M35 pectinase production indicated an adequate signal and the model can be used to navigate the design space. The following equation shows fitted quadratic model in terms of actual factors:

Pectinase = -175.34669 + 468.44025 * WB + 283.31450*CP + 5.51010*WB*CP - 151.41107*WB^2^ - 115.41191*CP^2^.

**Table B1. Fit summary plot for M35-pectinase production obtained using CCD:**

| 1. Sequential Model Sum of Squares [Type I] | | | | | | | |
| --- | --- | --- | --- | --- | --- | --- | --- |
| Source | Sum of Squares | DOF | Mean Square | F-value | *p*-value, Prob>F | Prediction | |
| Mean vs Total | 255000.00 | 1 | 255000.00 |  |  |  | |
| Linear vs Mean | 33599.79 | 2 | 16799.89 | 0.70 | 0.5236 |  | |
| 2FI vs Linear | 143.71 | 1 | 143.71 | 0.01 | 0.9442 |  | |
| **Quadratic vs 2FI** | **189900.00** | **2** | **94972.90** | **373.64** | **< 0.0001** | **Suggested** | |
| Cubic vs Quadratic | 1.12 | 2 | 0.56 | 0.00 | 0.9987 | Aliased | |
| Residual | 1269.81 | 3 | 423.27 |  |  |  | |
| Total | 480000.00 | 11 | 43637.14 |  |  |  | |
| 2. Lack of Fit Tests | | | | | | |  |
| Source | Sum of Squares | DOF | Mean Square | F-value | *p*-value, Prob>F |  | |
| Linear | 191200.00 | 6 | 31871.00 | 474.21 | 0.0021 |  | |
| 2FI | 191100.00 | 5 | 38216.46 | 568.63 | 0.0018 |  | |
| **Quadratic** | **1136.51** | **3** | **378.84** | **5.64** | **0.1544** | **Suggested** | |
| Cubic | 1135.39 | 1 | 1135.39 | 16.89 | 0.0544 | Aliased | |
| Pure Error | 134.42 | 2 | 67.21 |  |  |  | |
| 3. Model Summary Statistics | | | | | | |  |
| Source | Std. Deviation | R^2^ | Adjusted R^2^ | PredictedR^2^ | PRESS |  | |
| Linear | 154.66 | 0.1494 | -0.0633 | -0.3086 | 294400.00 |  | |
| 2FI | 165.28 | 0.1500 | -0.2143 | -0.6025 | 360500.00 |  | |
| **Quadratic** | **15.94** | **0.9944** | **0.9887** | **0.9627** | **8384.31** | **Suggested** | |
| Cubic | 20.57 | 0.9944 | 0.9812 | 0.6756 | 72967.57 |  | |

Table B2. ANOVA for M35-pectinase production obtained using CCD:

| ANOVA for Response Surface Quadratic Model | | | | | | |
| --- | --- | --- | --- | --- | --- | --- |
| Analysis of variance table [Partial sum of squares - Type III] | | | | | |  |
| Source | Sum of Squares | DOF | Mean Square | F-value | p-value, Prob>F |  |
| Model | 223700.00 | 5 | 44737.86 | 176 | < 0.0001 | significant |
| A-WB | 1968.36 | 1 | 1968.36 | 7.74 | 0.0388 |  |
| B-CP | 31631.43 | 1 | 31631.43 | 124.44 | 0.0001 |  |
| AB | 143.71 | 1 | 143.71 | 0.57 | 0.4860 |  |
| A^2^ | 153200.00 | 1 | 1.53E+05 | 602.69 | < 0.0001 |  |
| B^2^ | 89008.53 | 1 | 89008.53 | 350.17 | < 0.0001 |  |
| Residual | 1270.93 | 5 | 254.19 |  |  |  |
| Lack of Fit | 1136.51 | 3 | 378.84 | 5.64 | 0.1544 | not significant |
| Pure Error | 134.42 | 2 | 67.21 |  |  |  |
| Cor Total | 225000.00 | 10 |  |  |  |  |

**B.2. J208-pectinase production:**

Through the sequential analysis of the response surface for J208 pectinase production, it was observed that the quadratic model fits well with data. As shown in Table B3, the quadratic model was significant above 99.9 % confidence level with *p*-value 0.001. Similarly, the Lack of Fit F-value for quadratic model was 0.2793 suggesting that Lack of Fit was not significant, relative to the pure error and there is a 27.93% chance that a “Lack of Fit F-value” this large could occur due to noise.

The quadratic model values of correlation coefficient (R^2^), adjusted R^2^ and predicted R^2^ were 0.963, 0.927 and 0.774 respectively. Values of R^2^ and adjusted R^2^ were > 0.7. Predicted R^2^ was in reasonable agreement with the adjusted R^2^. PRESS value of 183000 was also suitable as it was least in comparison to other models. All these results from Table B3 indicated that the quadratic model was the most appropriate one for analysis of pectinase production data.

Therefore, analysis of variance (ANOVA) for J208 pectinase production response was performed using the quadratic model. The values of sum of squares, mean squares, F-value, and *p*-value for model, selected factors and their interactions are given in Table B4. *p*-values < 0.05 indicates that the model terms are significant. In this case the Model F-value of 7.7x10^5^ implies the model was highly significant with *p*-values < 0.002 (with confidance level > 99.8%) and there is only a 0.2% chance that a "Model F-Value" this large could occur due to noise. *p*-values less than 0.05 (at confidence level of 95%) indicates the significance of the model terms.

In this case A, B, AB, A^2^, B^2^ are significant model terms. *p*-values obtained for both individual factors, viz., A (wheat bran, WB) and B (citrus peel, CP) were 0.0166 and 0.0047 suggesting that CP had more significant effect on pectinase production than WB. The *p*-value of AB factor (0.9232) suggested that the interaction between WB and CP was significant for pectinase production at 95.00% confidence level also.

The adequate precision value (12.494) of signal to noise ratio for J208 pectinase production indicated an adequate signal and the model can be used to navigate the design space. The following equation shows fitted quadratic model in terms of actual factors:

Pectinase = -304.18615 + 846.60422*WB + 541.58813*CP + 3.57315*WB*CP -265.56470*WB^2^ - 220.63661*CP^2^.

Table B3. Fit summary plot for J208-pectinase obtained using CCD:

| 1. Sequential Model Sum of Squares [Type I] | | | | | | |
| --- | --- | --- | --- | --- | --- | --- |
| Source | Sum of Squares | DOF | Mean Square | F-value | *p*-value, Prob>F | Prediction |
| Mean vs Total | 1028000.00 | 1 | 1028000.00 |  |  |  |
| Linear vs Mean | 153400.00 | 2 | 76680.96 | 0.94 | 0.4282 |  |
| 2FI vs Linear | 60.43 | 1 | 60.43 | 0.00 | 0.9803 |  |
| **Quadratic vs 2FI** | **619900.00** | **2** | **309900.00** | **52.65** | **0.0004** | **Suggested** |
| Cubic vs Quadratic | 6655.87 | 2 | 3327.94 | 0.44 | 0.6808 | Aliased |
| Residual | 22780.38 | 3 | 7593.46 |  |  |  |
| Total | 1831000.00 | 11 | 166500.00 |  |  |  |
| 2. Lack of Fit Tests | | | | | |  |
| Source | Sum of Squares | DOF | Mean Square | F-value | *p*-value, Prob>F |  |
| Linear | 643600.00 | 6 | 107300.00 | 37.15 | 0.0264 |  |
| 2FI | 643500.00 | 5 | 128700.00 | 44.58 | 0.0221 |  |
| **Quadratic** | **23661.88** | **3** | **7887.29** | **2.73** | **0.2793** | **Suggested** |
| Cubic | 17006.01 | 1 | 17006.01 | 5.89 | 0.1360 | Aliased |
| Pure Error | 5774.36 | 2 | 2887.18 |  |  |  |
| 3. Model Summary Statistics | | | | | |  |
| Source | Std. Deviation | R^2^ | Adjusted R^2^ | PredictedR^2^ | PRESS |  |
| Linear | 284.91 | 0.1910 | -0.0112 | -0.2505 | 1004000.00 |  |
| 2FI | 304.56 | 0.1911 | -0.1555 | -0.4419 | 1157000.00 |  |
| **Quadratic** | **76.73** | **0.9633** | **0.9267** | **0.7742** | **181300.00** | **Suggested** |
| Cubic | 87.14 | 0.9716 | 0.9054 | -0.3720 | 1101000.00 | Aliased |

Table B4. ANOVA for J208-pectinase production obtained using CCD:

| ANOVA for Response Surface Quadratic Model | | | | | | |
| --- | --- | --- | --- | --- | --- | --- |
| Analysis of variance table [Partial sum of squares - Type III] | | | | | |  |
| Source | Sum of Squares | DOF | Mean Square | F-value | p-value, Prob>F |  |
| Model | 773300.00 | 5 | 154700.00 | 26.27 | 0.0013 | Significant |
| A-WB | 15410.44 | 1 | 15410.44 | 2.62 | 0.0166 |  |
| B-CP | 138000.00 | 1 | 138000.00 | 23.43 | 0.0047 |  |
| AB | 60.43 | 1 | 60.43 | 0.01 | 0.9232 |  |
| A^2^ | 471300.00 | 1 | 471300.00 | 80.05 | 0.0003 |  |
| B^2^ | 325300.00 | 1 | 325300.00 | 55.26 | 0.0007 |  |
| Residual | 29436.25 | 5 | 5887.25 |  |  |  |
| Lack of Fit | 23661.88 | 3 | 7887.29 | 2.73 | 0.2793 | not significant |
| Pure Error | 5774.36 | 2 | 2887.18 |  |  |  |
| Cor Total | 802700.00 | 10 |  |  |  |  |

**C.1. Model diagnostics:**

During analysis of the designed experiment and received responses, the Design expert software provided different diagnostic plots which helped to study the effect of selected experimental variables on responses. Few of such plots like (I) Normal probability plot of residuals, (II) Plot of studentized residuals versus predicted residuals and (III) Plot of actual versus predicted values were used to diagnose the statistical properties of the model and its adequacy, which is an important part of data analysis.

**C.1.1. Normal probability plot of residuals:**

Normal probability plot of residuals is the most important part of the diagnostics. These plots indicate whether the residuals follow a normal distribution, i.e., the residual plots will follow a straight line. The normality assumptions for residuals of individual run for xylanase and pectinase production responses from each of the isolates when plotted, were found to be distributed around a straight line suggesting that the quadratic model fits well for both the responses from each isolate as shown in Figure C1.1 (A-D).

**C.1.2. Plots of studentized residuals versus predicted values:**

This plot tests the assumption of constant variance. As shown in Figure C1.2 (A-D), the patterns of the plots for xylanase and pectinase production responses showed random distribution of studentized residuals in all the runs, indicated that the assumption of constant variance obtained using the quadratic model was true.

**C.1.3. Plot of actual versus predicted values:**

This plot shows point of the actual response values versus the predicted response values. A pattern of the plot of the actual values versus the predicted values for xylanase and pectinase production response were distributed on or around the line and indicated that the predicted data points and actual data points matched and the quadratic model fits well for each response Figure C1.3 (A-D).

A)
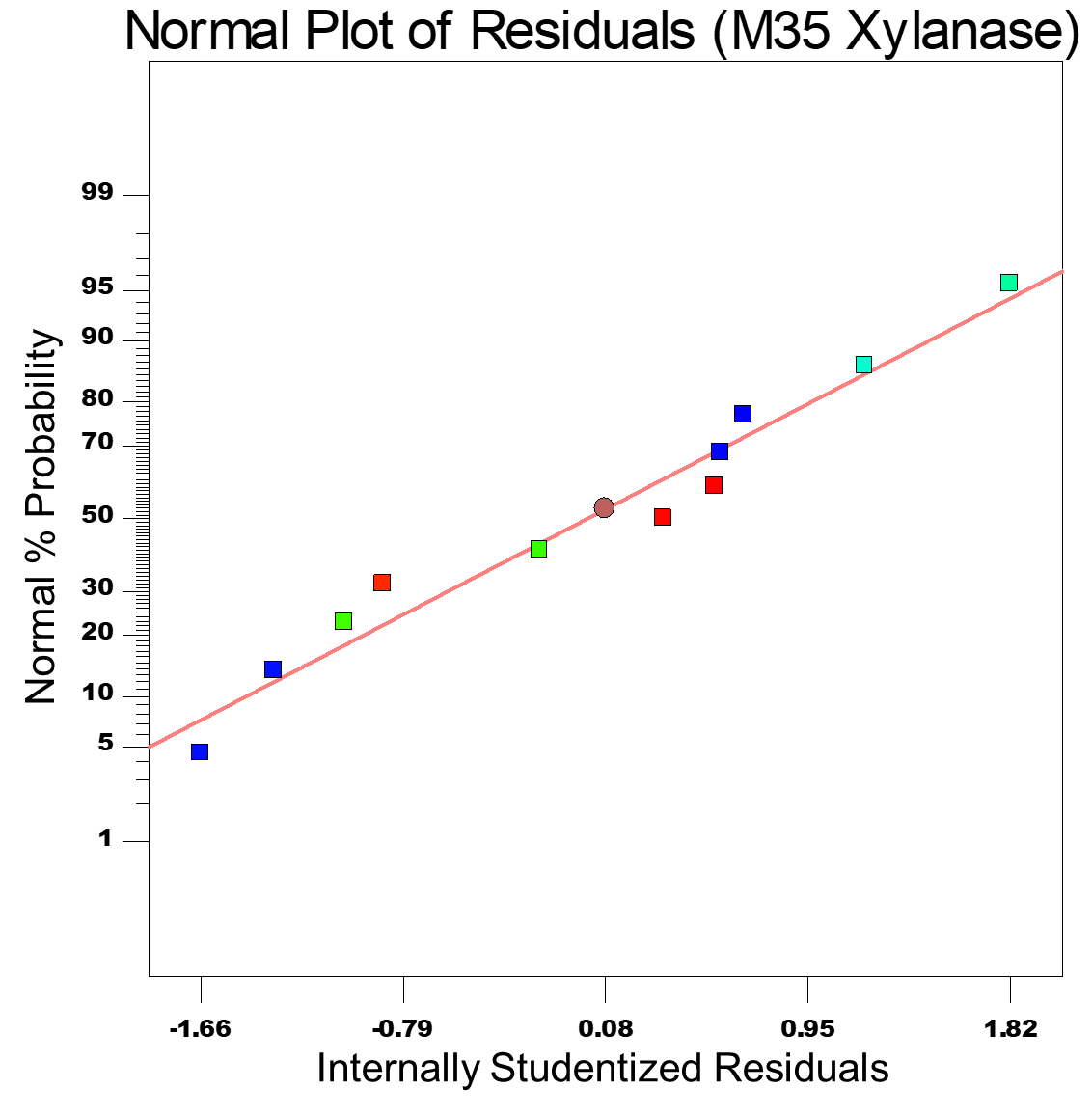
 B)
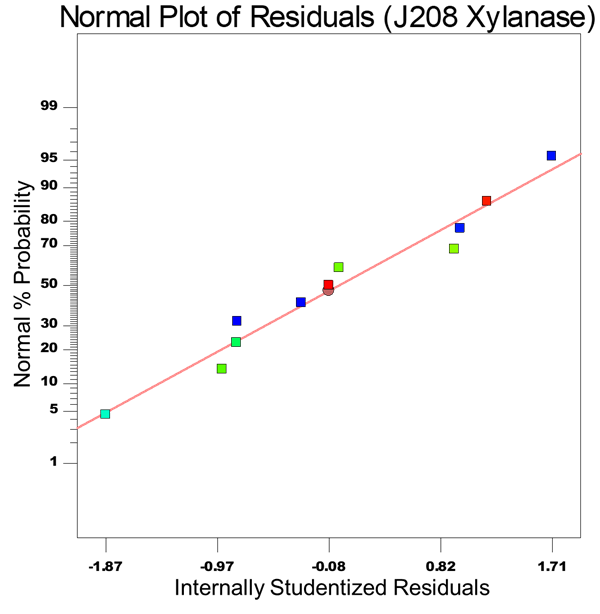


C)
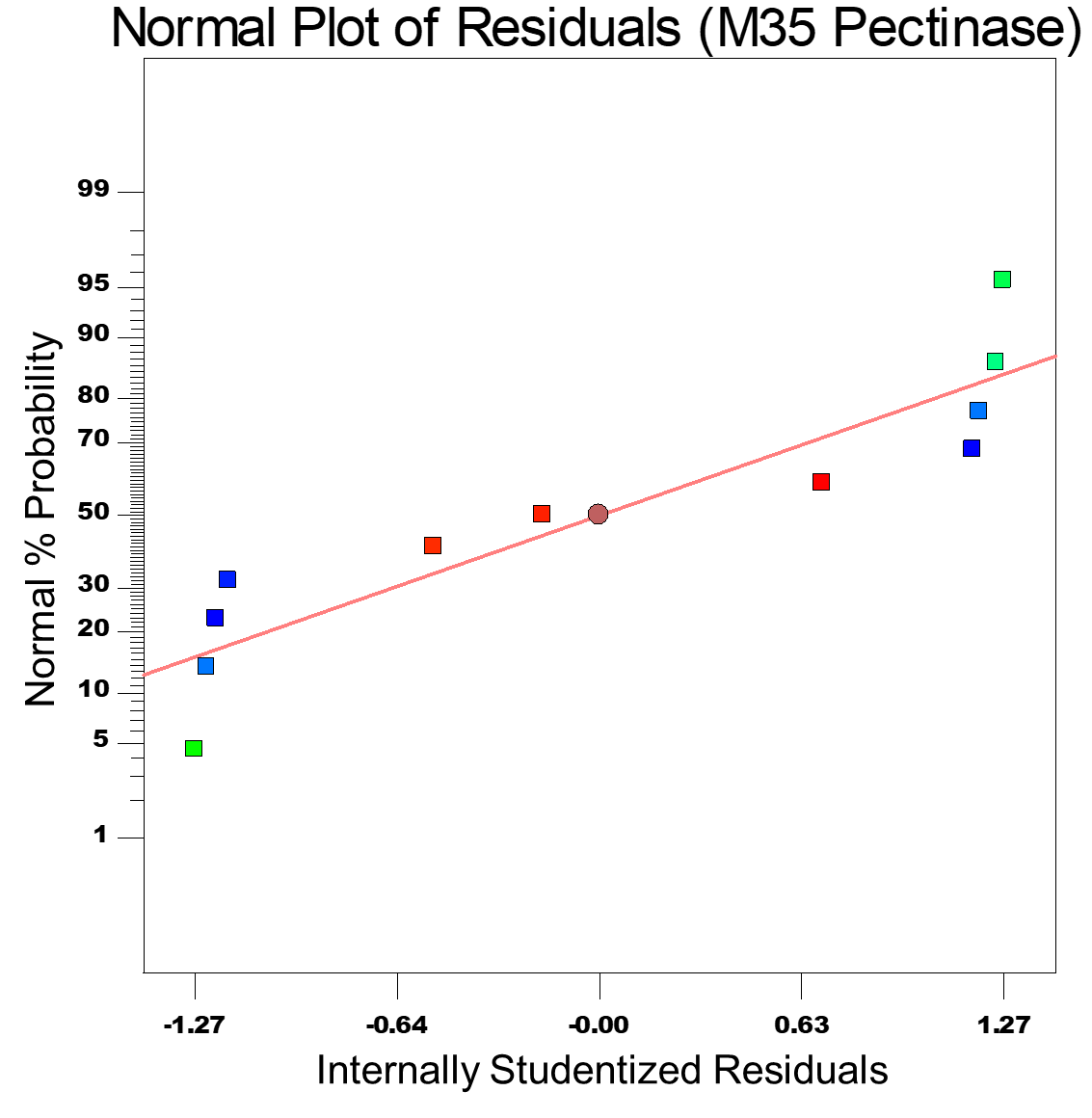
D)
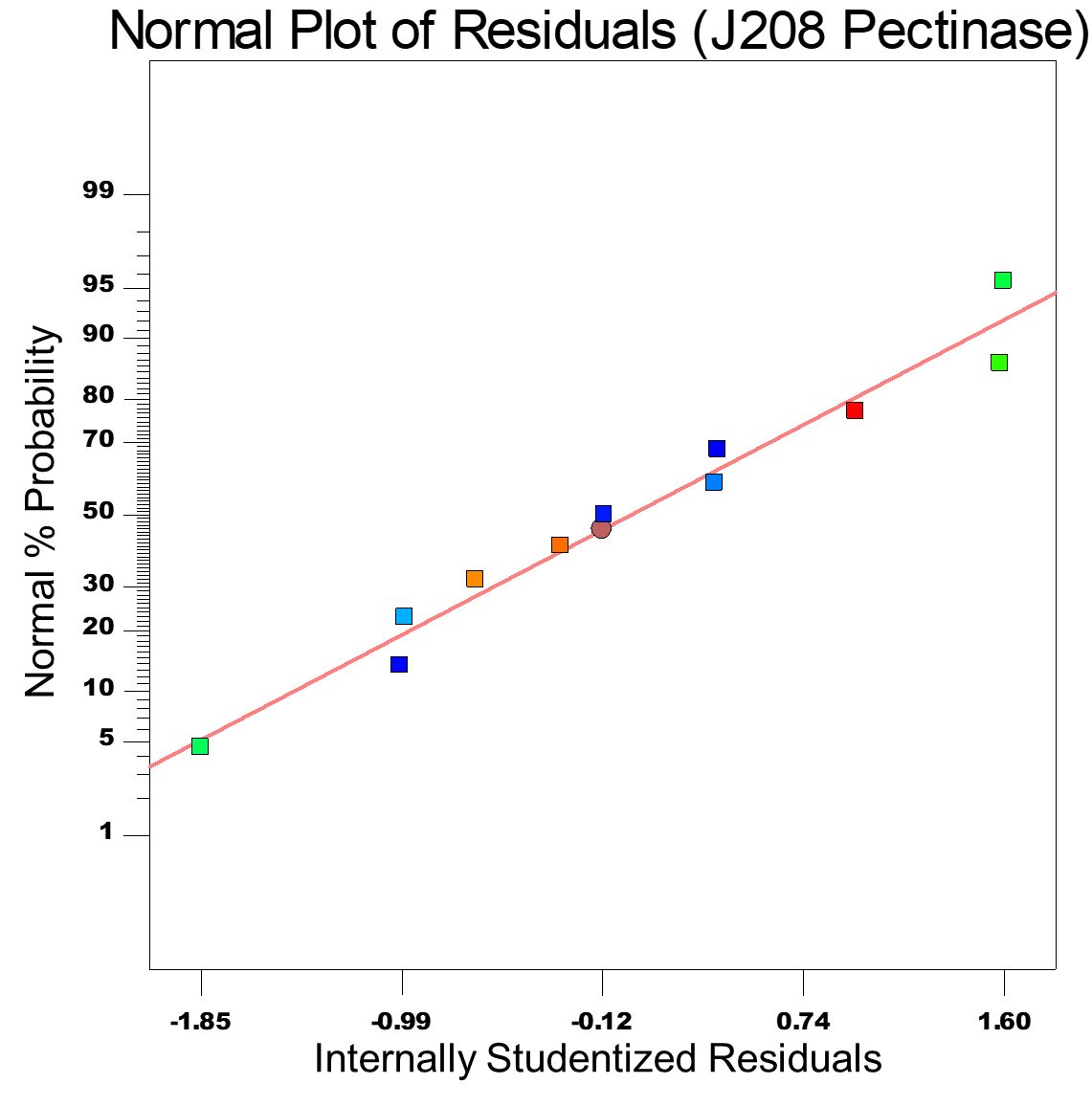


Figure C1.1. Normal probability plot of residuals for xylanase and pectinase production responses obtained using CCD from *Bacillus* spp. isolates:

**(A-B)**: Normal probability plot of residuals for xylanase production response obtained from **(A)** *B. safensis* M35 and **(B)** *B. altitudinis* J208: **(C-D)**: Normal probability plot of residuals for pectinase production response obtained from **(C)** *B. safensis* M35 and **(D)** *B. altitudinis* J208.

A)
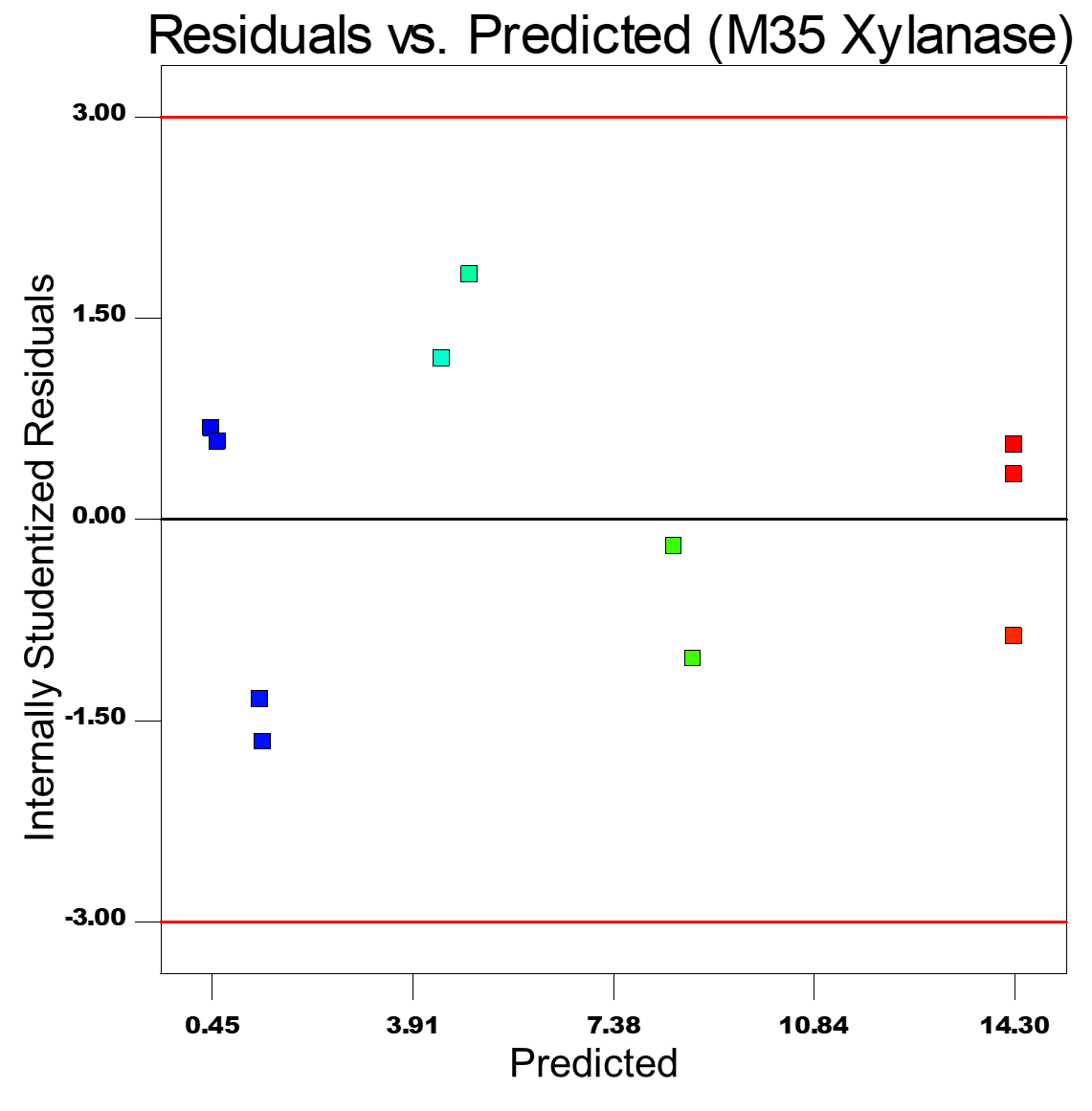
 B)
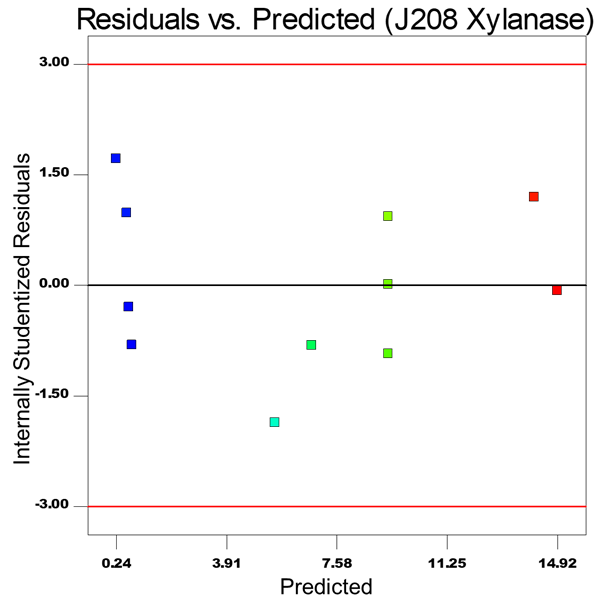


C)
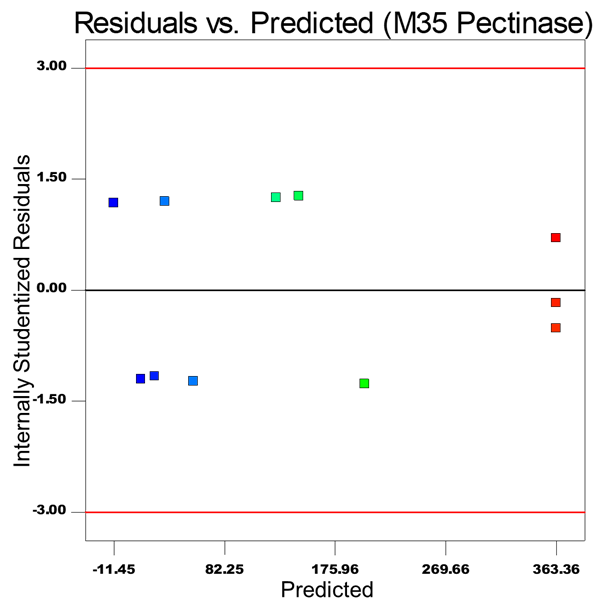
 D)
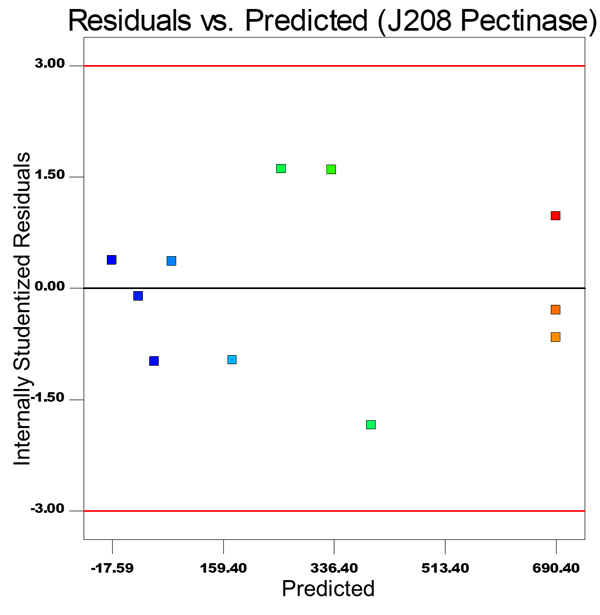


Figure C1.2. Plot of studentized residuals versus predicted values for xylanase and pectinase production responses obtained using CCD from *Bacillus* spp. isolates:

**(A-B)**: Plot of studentized residuals versus predicted values for xylanase production response obtained from **(A)** *B. safensis* M35 and **(B)** *B. altitudinis* J208; **(C-D)**: Plot of studentized residuals versus predicted values for pectinase production response obtained from **(C)** *B. safensis* M35 and **(D)** *B. altitudinis* J208.

A)
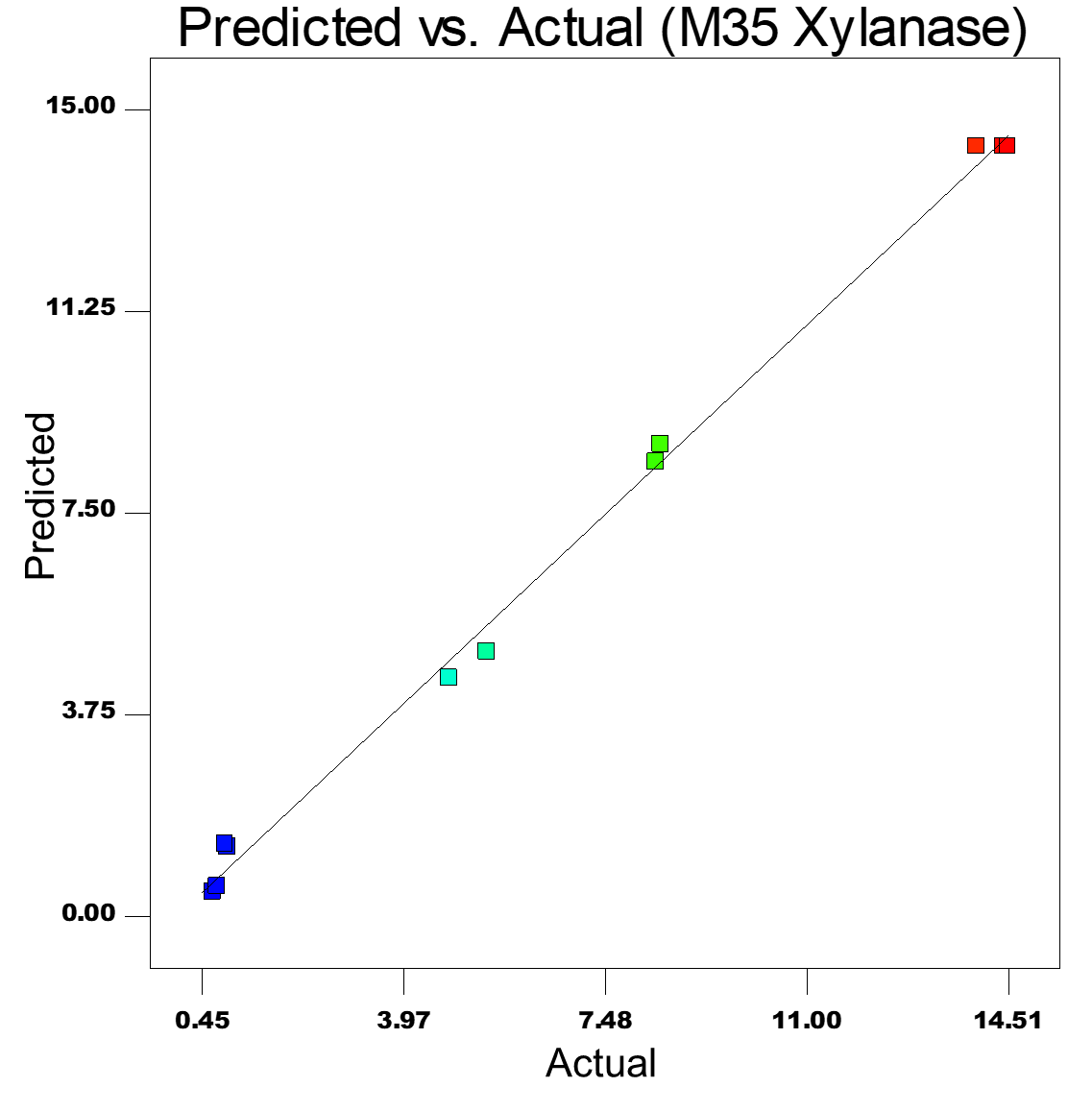
 B)
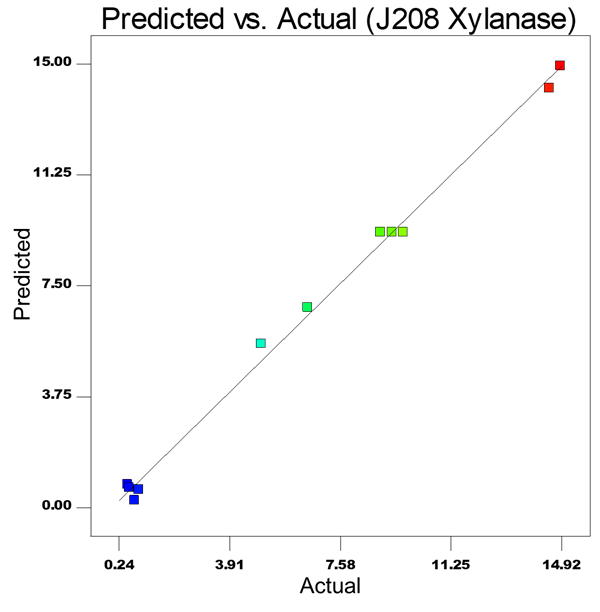


C)
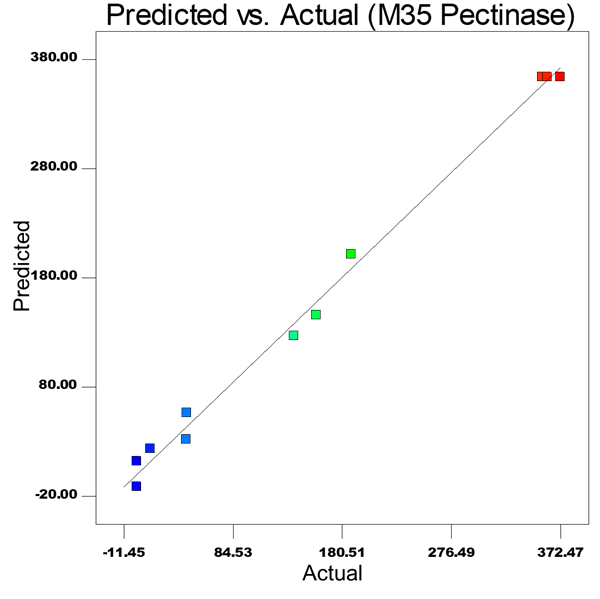
D)
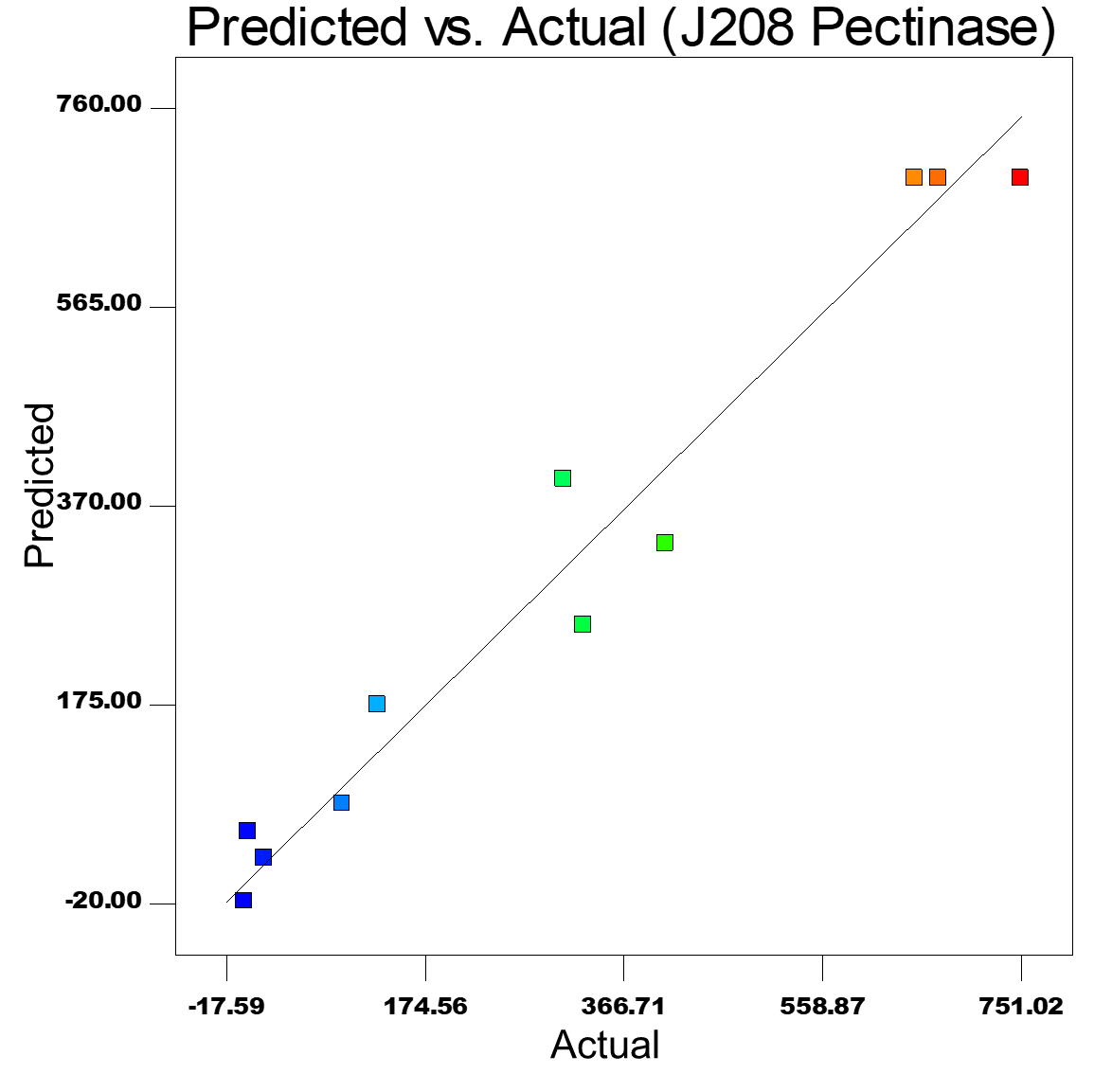


Figure C1.3: Plot of actual versus predicted values for xylanase and pectinase production responses obtained using CCD from *Bacillus* spp. isolates:

**(A-B)**: Plot of actual versus predicted values for xylanase production response obtained from **(A)** *B. safensis* M35 and **(B)** *B. altitudinis* J208; **(C-D)**: Plot of actual versus predicted values for pectinase production response obtained from **(C)** *B. safensis* M35 and **(D)** *B. altitudinis* J208.

**D.1. Model graphs:**

As diagnosis of residuals revealed no statistical problem and showed that the quadratic models were suitable, further response surface plots of xylanase and pectinase production response for each isolate were generated.

**D1.1. Xylanase production response:**

The quadratic model suited well for xylanase production response. The one factor effect plots representing the xylanase production over changes in independent variable A-WB and B-CP are shown for each of the *Bacillus* isolate, i.e., M35 and J208 in Figure D1.1 (A-D). It was visible that increase of WB and CP concentrations up to certain level, positively affected xylanase production while beyond that concentration, they negatively affect the xylanase production response by *B. safensis* M35 (A, B). While the plots C suggested the moderate positive effect of WB on xylanase production response, negative effect of CP on xylanase production response was exhibited by plots D for *B. altitudinis* J208. While. These results are in accordance with the results present in Table, A2 and A4 where it was noted that the CP had more significant effect on xylanase production response than WB.

**D1.2. Pectinase production response:**

The quadratic model suited well for pectinase production response. The one factor effect plots representing the pectinase production over changes in independent variables A-WB and B-CP are shown for each of the *Bacillus* isolate, i.e., M35 and J208 in Figure D1.2 (A-D). It was visible that increase of WB and CP concentrations up to certain level, positively affected pectinase production while beyond that concentration, they negatively affect the xylanase production response by isolates *B. safensis* M35, B. altitudinis J208. These results are in accordance with the results present in Table, B2 and B4 where it was noted that the CP had more significant effect on pectinase production response than WB.

A)
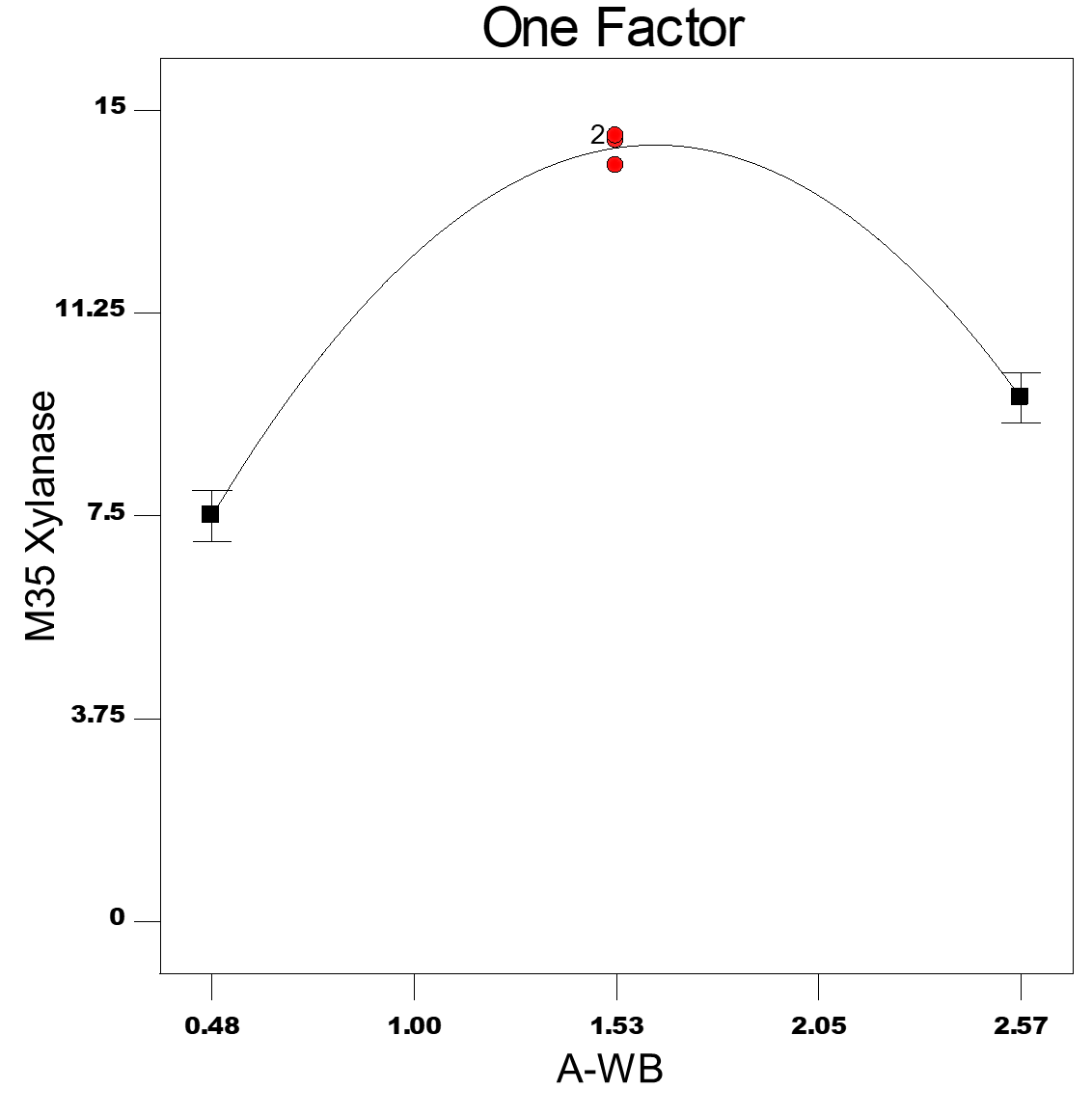
B)
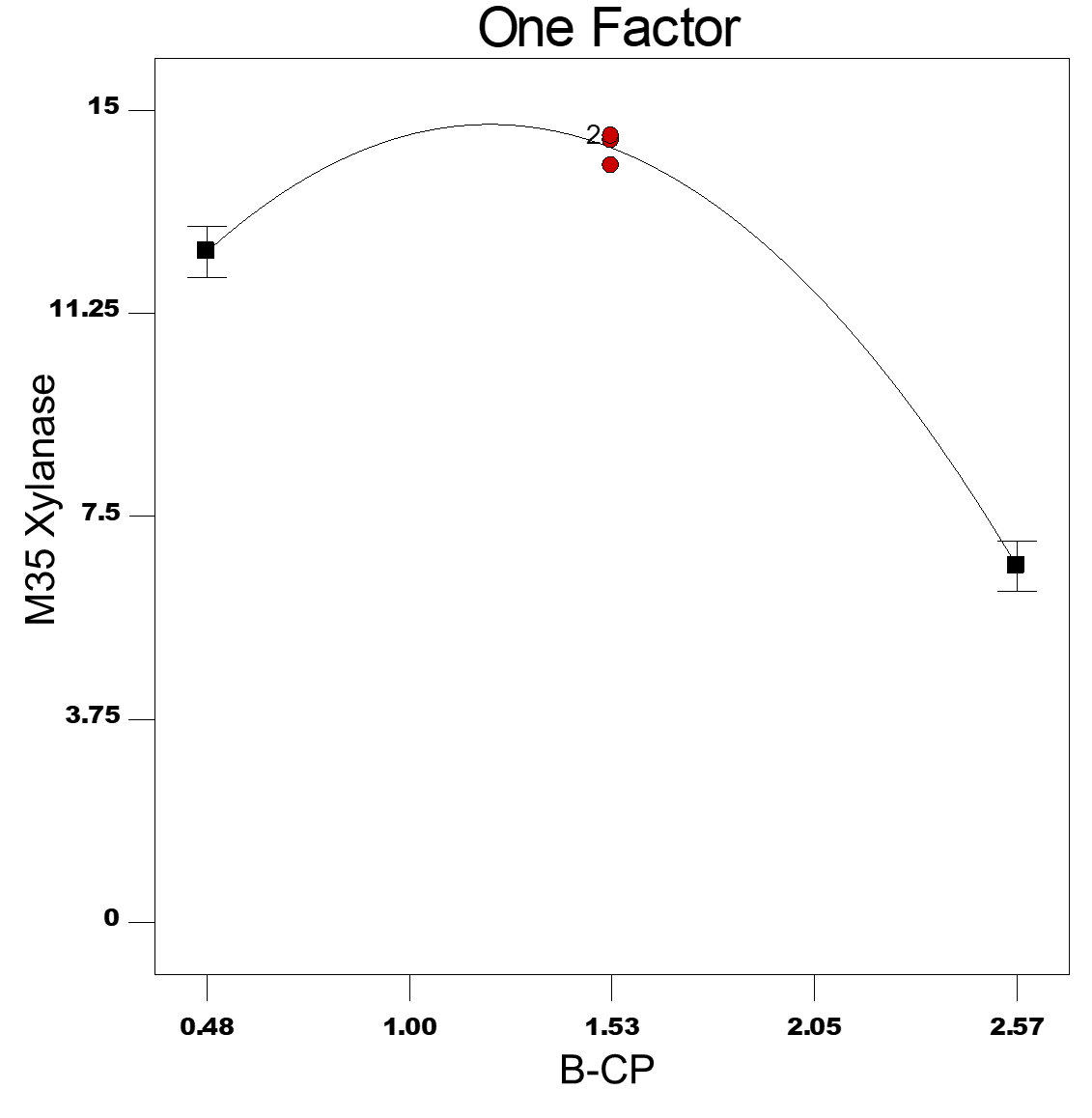


C)
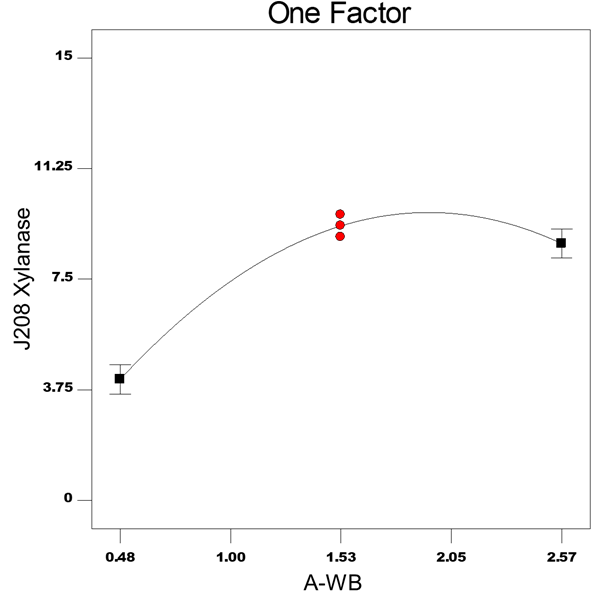
 D)
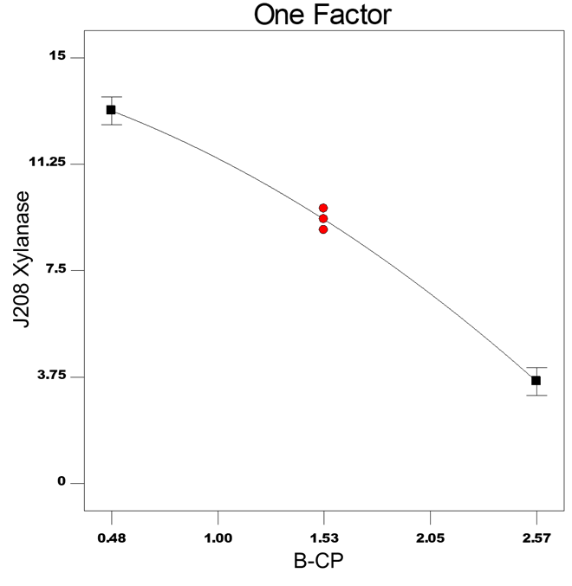


**Figure D1.1. One factor plots showing the effect of WB and CP on xylanase production response from *Bacillus* isolates.**

**(A, B)**: Effect of **(A)** WB and **(B)** CP on xylanase production response by *B. safensis* M35; **(C, D)**: Effect of **(C)** WB and **(D)** CP on xylanase production response by *B. altitudinis* J208.

A)
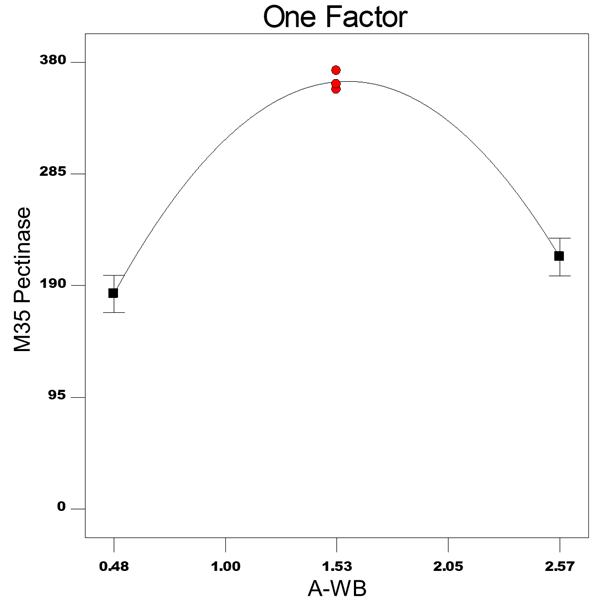
B)
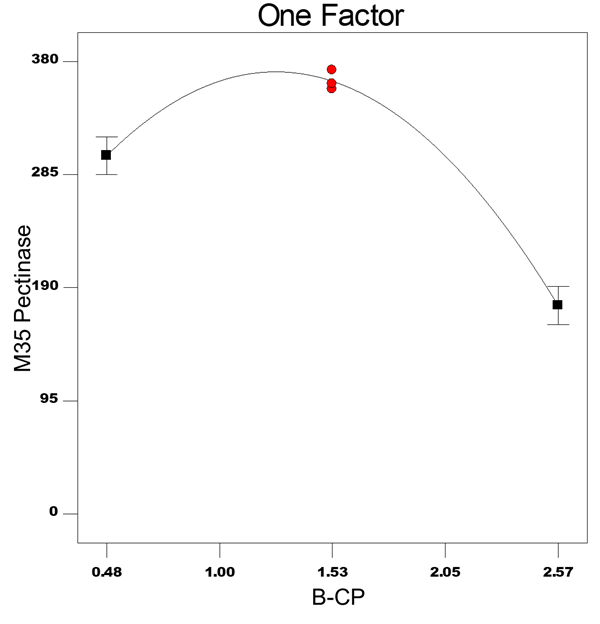


C)
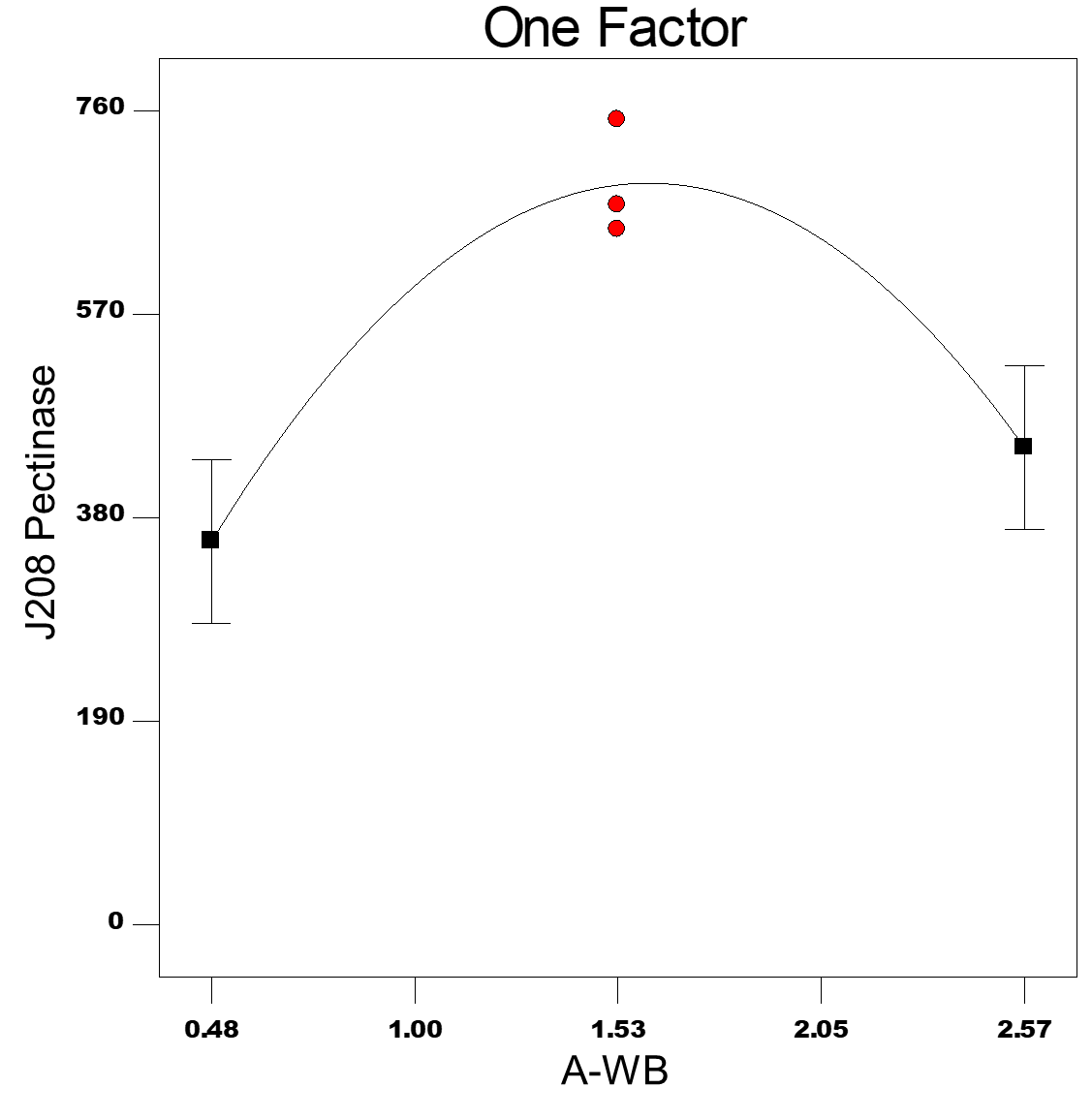
 D)
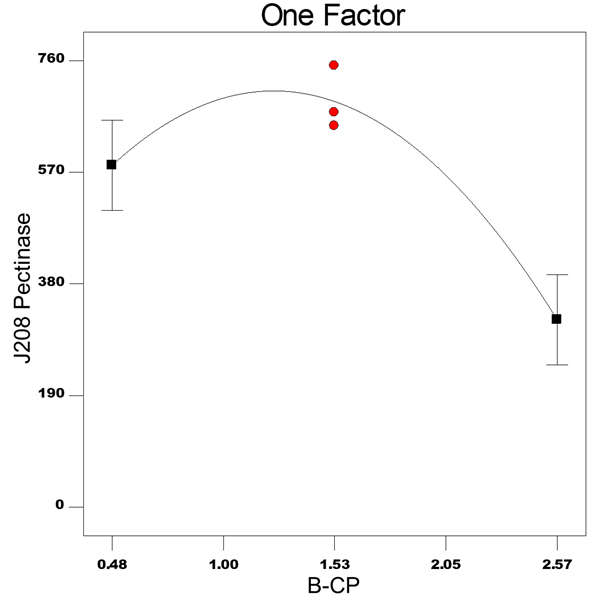


Figure D1.2. One factor plots showing the effect of WB and CP on pectinase production response from *Bacillus* isolates.

**(A, B):** Effect of **(A)** WB and **(B)** CP on pectinase production response by *B. safensis* M35; **(C, D)**: Effect of **(C)** WB and **(D)** CP on pectinase production response by *B. altitudinis* J208.
